# Durable spike-specific T cell responses after different COVID-19 vaccination regimens are not further enhanced by booster vaccination

**DOI:** 10.1126/sciimmunol.add3899

**Published:** 2022-12-16

**Authors:** Yacine Maringer, Annika Nelde, Sarah M. Schroeder, Juliane Schuhmacher, Sebastian Hörber, Andreas Peter, Julia Karbach, Elke Jäger, Juliane S. Walz

**Affiliations:** ^1^ Department of Peptide-based Immunotherapy, University and University Hospital Tübingen, Tübingen, Germany.; ^2^ Institute for Cell Biology, Department of Immunology, University of Tübingen, Tübingen, Germany.; ^3^ Cluster of Excellence iFIT (EXC2180) “Image-Guided and Functionally Instructed Tumor Therapies”, University of Tübingen, Tübingen, Germany.; ^4^ Department of Otorhinolaryngology, Head and Neck Surgery, University Hospital Tübingen, Tübingen, Germany.; ^5^ Institute for Clinical Chemistry and Pathobiochemistry, Department for Diagnostic Laboratory Medicine, University Hospital Tübingen, Tübingen, Germany.; ^6^ Department of Oncology and Hematology, Krankenhaus Nordwest, Frankfurt, Germany.; ^7^ Clinical Collaboration Unit Translational Immunology, German Cancer Consortium (DKTK), Department of Internal Medicine, University Hospital Tübingen, Tübingen, Germany.

## Abstract

Several COVID-19 vaccines are approved to prevent severe disease outcome after SARS-CoV-2 infection. Whereas induction and functionality of antiviral antibody response are largely studied, the induction of T cells upon vaccination with the different approved COVID-19 vaccines is less studied. Here, we report on T cell immunity 4 weeks and 6 months after different vaccination regimens and 4 weeks after an additional booster vaccination in comparison with SARS-CoV-2 T cell responses in convalescents and prepandemic donors using interferon-gamma ELISpot assays and flow cytometry. Increased T cell responses and cross-recognition of B.1.1.529 Omicron variant–specific mutations were observed ex vivo in mRNA- and heterologous-vaccinated donors compared with vector-vaccinated donors. Nevertheless, potent expandability of T cells targeting the spike protein was observed for all vaccination regimens, with frequency, diversity, and the ability to produce several cytokines of vaccine-induced T cell responses comparable with those in convalescent donors. T cell responses for all vaccinated donors significantly exceeded preexisting cross-reactive T cell responses in prepandemic donors. Booster vaccination led to a significant increase in anti-spike IgG responses, which showed a marked decline 6 months after complete vaccination. In contrast, T cell responses remained stable over time after complete vaccination with no significant effect of booster vaccination on T cell responses and cross-recognition of Omicron BA.1 and BA.2 mutations. This suggested that booster vaccination is of particular relevance for the amelioration of antibody response. Together, our work shows that different vaccination regimens induce broad and long-lasting spike-specific CD4^+^ and CD8^+^ T cell immunity to SARS-CoV-2.

## Teaser

Durable T cell responses with Omicron cross-recognition are observed after complete and booster vaccination with different COVID-19 vaccines.

## INTRODUCTION

During the coronavirus disease 2019 (COVID-19) pandemic, caused by the severe acute respiratory syndrome coronavirus 2 (SARS-CoV-2), several vaccines have been successfully developed, reducing transmission and preventing billions of people from severe disease outcome (*1*–*4*). Among the currently approved COVID-19 vaccines, the ChAdOx1 nCoV-19 adenovirus-based vector vaccine ChAdOx1, the human adenovirus type 26 (Ad26)–based vector vaccine Ad26.COV2.S, and the two mRNA vaccines BNT162b2 and mRNA-1273 are the most widely used in Europe and North America (*5*–*7*). Vaccination schedules comprise two doses of ChAdOx1, BNT162b2, and mRNA-1273 and one dose of Ad26.COV2.S for complete vaccination status (*1*–*4*). After reports of thromboembolic events after ChAdOx1 vaccination (*8*), several European governments recommended completing vaccination with an mRNA vaccine after the first dose of ChAdOx1 (heterologous vaccination). To overcome waning vaccine immunity over time (*9*), the administration of an additional booster vaccine dose was approved in many countries 3 to 6 months after completion of vaccination (*10*).

COVID-19 vaccination induces both humoral immunity, mediated by B cell–derived antibodies, and cellular immunity, mediated by T cells (*2*). Although it is undisputed that neutralizing antibodies provide the first line of antiviral defense (*11*, *12*), T cell immunity is crucial to combat acute SARS-CoV-2 infection and for the development of long-term immunity (*13*). Whereas antibody titers tend to wane quickly and show limited neutralizing activity to newly arising variants of concern (VOCs), T cell memory is largely conserved against VOCs after prior SARS-CoV-2 infection (*14*, *15*).

So far, research on SARS-CoV-2 vaccine-induced immunity is largely focused on anti-spike antibody titers and their ability to neutralize virus particles (*16*). Spike-specific T cell responses induced upon different vaccination regimens are studied to a lesser extent, with first reports showing the induction of both CD4^+^ and CD8^+^ T cell responses after complete vaccination with different vaccination regimens. Moreover, T cell responses are shown to be largely conserved against different SARS-CoV-2 variants, including early B.1.1.529 (Omicron) variants now dominant globally (*12*, *17*).

In this work, we provided an analysis of spike-specific T cell responses and their cross-recognition of B.1.1.529 Omicron BA.1 and BA.2 variant-specific mutations after complete vaccination with mRNA, vector, and heterologous vaccine regimens in comparison with COVID-19 convalescents and prepandemic donors. In addition, we provided insight on the effects of a third mRNA booster vaccination after homologous and heterologous vaccination regimens on T cell and antibody immunity.

## RESULTS

### SARS-CoV-2 spike–specific T cell responses after complete vaccination with different vaccination regimens

**Fig. 1.**
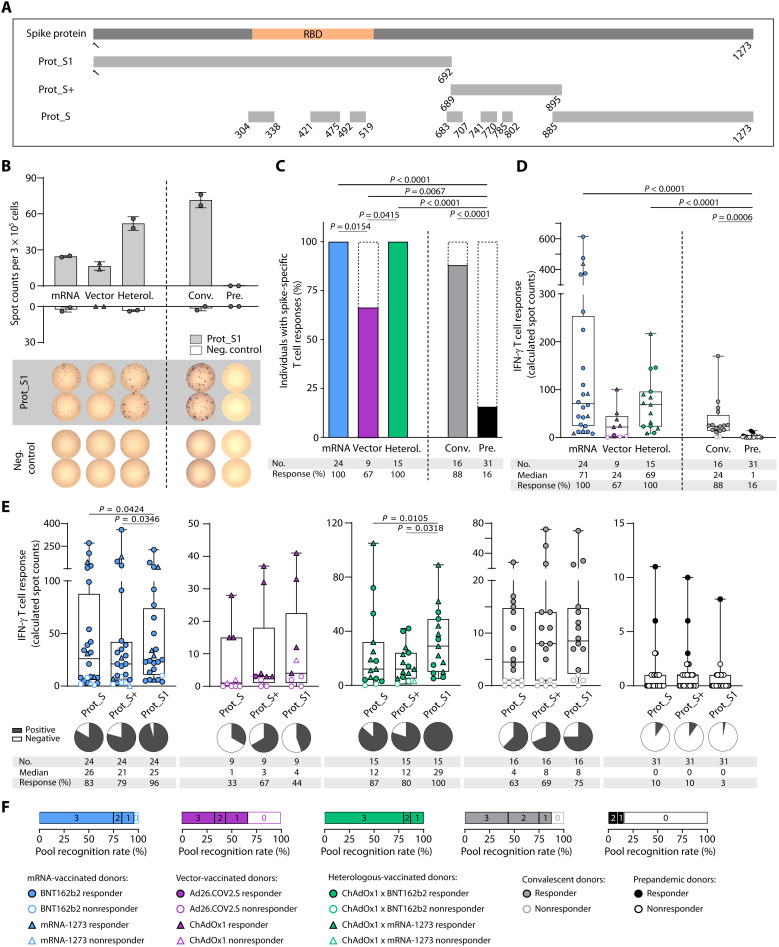
Ex vivo immune responses to SARS-CoV-2 spike protein peptide pools after complete vaccination. (**A**) Schematic depiction of the SARS-CoV-2 spike protein and protein section coverage by the peptide pools (Prot_S1, Prot_S+, and Prot_S) used for immunogenicity testing. (B to F) Ex vivo T cell responses after complete vaccination [two doses of BNT162b2, mRNA-1273, or ChAdOx1; one dose of Ad26.COV2.S; or one dose of the vector vaccine ChAdOx1 followed by one dose of an mRNA vaccine for heterologous (Heterol.) vaccine regimens] compared with COVID-19 convalescents (Conv.) and prepandemic (Pre.) donors were assessed by IFN-γ ELISpot assays 3 to 12 weeks (median, 4 weeks) after the last vaccine dose (sample collection after complete vaccination; Table 1). (**B**) Representative example of ex vivo IFN-γ T cell responses to the Prot_S1 peptide pool compared with a negative (neg.) control peptide showing duplicates for one donor of each cohort. (**C** and **D**) Percentage of individuals with ex vivo IFN-γ ELISpot T cell responses (C) and intensities of IFN-γ T cell responses in terms of calculated spot counts against the spike-specific peptide pools (D) after mRNA, vector, or heterologous vaccination compared with convalescents and prepandemic donors (summarized responses against the three spike-specific peptide pools). (**E**) Intensities of ex vivo IFN-γ T cell responses shown for the distinct spike protein peptide pools. (**F**) Proportion of individuals (cohorts as indicated by color code) with responses to all three, two, one, or none of the spike peptide pools. Responders are represented by colored symbols, and nonresponders are represented by clear symbols. Symbol shapes indicate the different vaccine products received by the donors. (D and E) Box plots represent the median with the 25th and 75th percentiles with minimum and maximum whiskers. (C) Fisher’s exact test was used. (D) Kruskal-Wallis test was used. (E) Friedman test was used. If *P* values are not shown, then results were not significant. RBD, receptor binding domain; No., number.

To assess spike-specific T cell responses after complete vaccination (two doses of BNT162b2, mRNA-1273, or ChAdOx1; one dose of Ad26.COV2.S; or one dose of vector vaccine ChAdOx1 followed by one dose of an mRNA vaccine for heterologous vaccine regimens), we performed interferon-γ (IFN-γ) enzyme-linked immunospot (ELISpot) assays 3 to 12 weeks (median, 4 weeks) after the complete vaccination dose (Table 1). Results were obtained using three different peptide pools covering various parts of the spike protein, with Prot_S1 covering the complete N-terminal S1 domain, Prot_S+ covering part of the C-terminal S2 domain, and Prot_S comprising selected immunodominant sequence domains (Fig. 1, A and B). Asymptomatic infections during the study period were excluded by testing for nucleocapsid antibodies (fig. S1).

**Table 1.** Characteristics of healthy volunteer cohorts after heterologous-, mRNA-, or vector-based vaccination. The mRNA-based vaccine cohort includes healthy volunteers vaccinated two (complete vaccination) or three times (booster vaccination) with either mRNA-1273 or BNT162b2. Donors of the vector-based vaccine either received two doses of ChadOx1 (complete vaccination) or one dose of Ad26.COV2.S (complete vaccination). The heterologous vaccination group received one dose of ChadOx1 followed by one (complete vaccination) or two doses (booster vaccination) of either mRNA-1273 or BNT162b2. For mRNA- and heterologous-vaccinated donors, we analyzed two different time points after complete vaccination, the time point of complete vaccination 3 to 12 weeks after complete vaccination and the time point 6 months after complete vaccination (21 to 32 weeks after complete vaccination). Figures 1 to 3 show T cell responses after complete vaccination, and analysis over different time points is shown in Fig. 4. Figure 4 includes the identical results for the time point of complete vaccination for mRNA- and heterologous-vaccinated donors as shown in Figs. 1 to 3. Experiments were not always conducted with all donors, depending on available cell numbers. *n*, number; n.a., not applicable; CV, complete vaccination.

	mRNA vaccine cohort	Vector vaccine cohort	Heterologous vaccination cohort
**Number of donors**	35	10	17
**Age (years)**			
Median	38	n.a.	31
Range	25–71	n.a.	24–52
**Sex [*n* (%)]**			
Female	19 (54.3)	n.a.	11 (64.7)
Male	16 (45.7)	n.a.	6 (35.3)
**Comorbidities [*n* (%)]**			
High blood pressure	3 (12.5)	n.a.	1 (5.9)
Cardiovascular disease	1 (4.2)	n.a.	1 (5.9)
Blood sugar disorder	2 (8.3)	n.a.	0 (0.0)
Chronic lung disease	0 (0.0)	n.a.	1 (5.9)
Cancer disease	1 (4.2)	n.a.	0 (0.0)
n.a.	11	n.a.	0
**Vaccination schemes (CV)**			
BNT162b2 x BNT162b2	20 (83.3)		
mRNA-1273 x mRNA-1273	4 (16.7)		
ChadOx1 x ChadOx1		6 (60.0)	
Ad26.COV2.S		4 (40.0)	
ChadOx1 x BNT162b2			7 (46.7)
ChadOx1 x mRNA-1273			8 (53.3)
**Time points**			
**Prevaccination**			
Donors	–	–	9
**After the first vaccination**			
Donors	–	–	8
Weeks after vaccination			
Median	–	–	10
Range	–	–	9–10
**After complete vaccination**			
Donors	24	10	15
Weeks after vaccination			
Median	3	4	6
Range	3–10	3–8	3–12
Awareness of side effects [*n* (%)]			
Yes	9 (69.2)	n.a.	5 (50.0)
No	4 (30.8)	n.a.	5 (50.0)
n.a.	11	n.a.	5
**6 months after complete vaccination**			
Donors	11	–	17
Weeks after vaccination			
Median	26	–	26
Range	21–32	–	24–28
**After booster vaccination**			
Donors	13	–	17
Weeks after vaccination			
Median	4	–	4
Range	2–7	–	3–6
Awareness of side effects [*n* (%)]			
Yes	2 (18.2)	–	6 (35.3)
No	9 (81.8)	–	11 (64.7)
n.a.	2	–	0

**Fig. 2.**
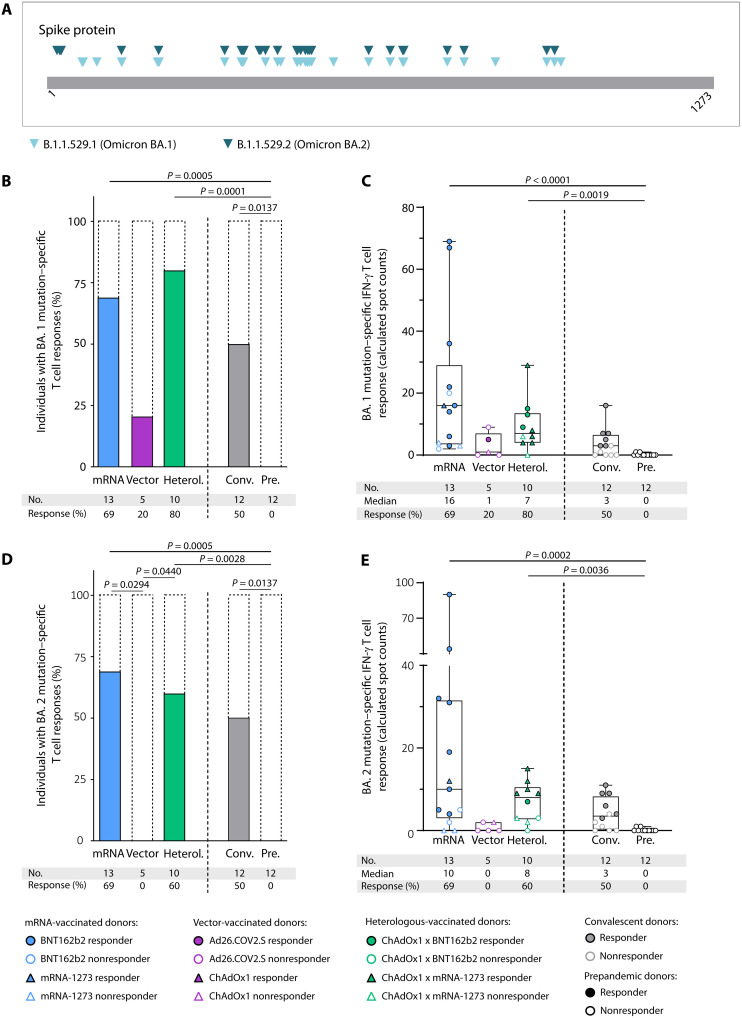
Ex vivo IFN-γ responses to SARS-CoV-2 BA.1 and BA.2 mutation pools. (**A**) Overview of variant-defining mutations in the spike protein described for the different VOCs. (B to E) Variant mutation–specific T cell responses after complete vaccination (two doses of BNT162b2, mRNA-1273, and ChAdOx1 or one dose of Ad26.COV2.S; for heterologous (Heterol.) vaccine regimens, one dose of the vector vaccine ChAdOx1 followed by one dose of an mRNA vaccine) were assessed by IFN-γ ELISpot assays. (**B**) Percentage of individuals with BA.1 mutation pool–specific ex vivo IFN-γ T cell responses and (**C**) intensities of IFN-γ T cell responses in terms of calculated spot counts after mRNA, vector, or heterologous vaccination compared with COVID-19 convalescents (Conv.) and prepandemic (Pre.) donors. (**D**) Percentage of individuals with BA.2 mutation pool–specific ex vivo IFN-γ T cell responses and (**E**) intensities of IFN-γ T cell responses in terms of calculated spot counts. Responders are represented by colored symbols, and nonresponders are represented by clear symbols. Symbol shapes indicate the different vaccine products received by the donors. (C and E) Box plots represent the median with the 25th and 75th percentiles with minimum and maximum whiskers. (B and D) Fisher’s exact test was used. (C and E) Kruskal-Wallis test was used. If *P* values are not shown, then results were not significant.

**Fig. 3.**
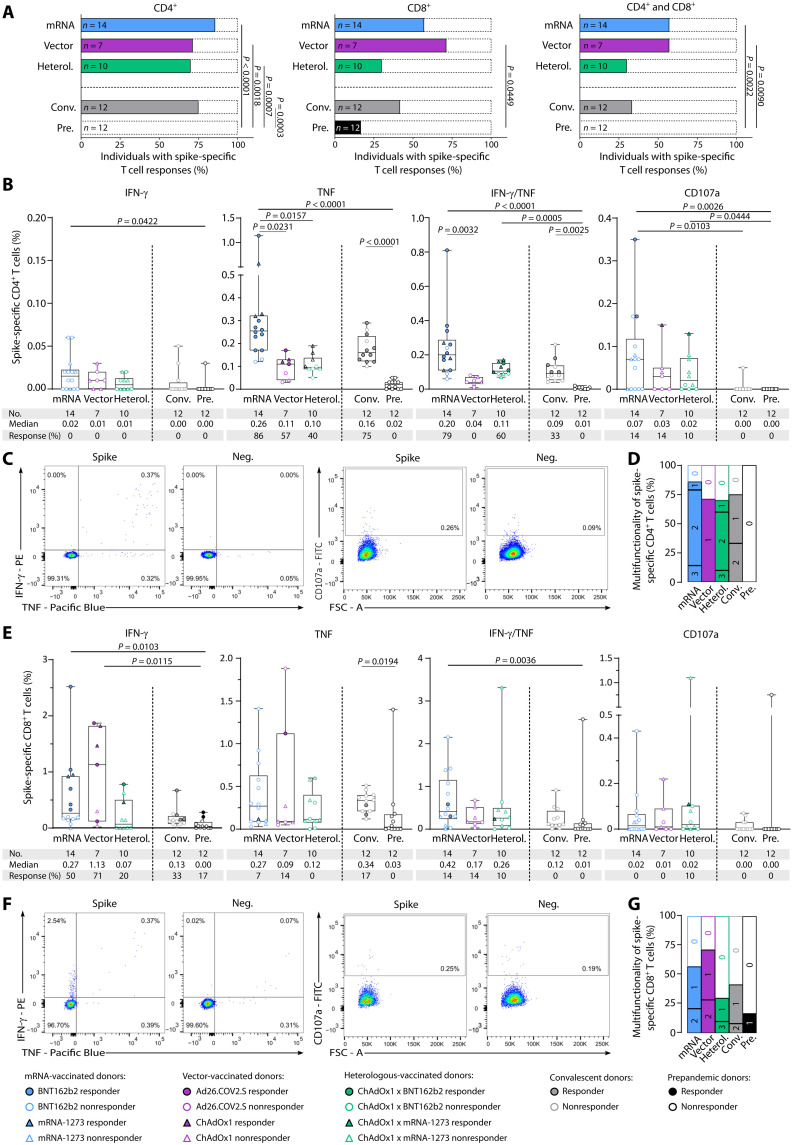
Ex vivo characterization of spike-specific T cell responses after complete vaccination. Spike-specific T cell responses after complete vaccination (two doses of BNT162b2, mRNA-1273, or ChAdOx1; one dose of Ad26.COV2.S; or one dose of the vector vaccine ChAdOx1 followed by one dose of an mRNA vaccine for heterologous (Heterol. vaccine regimens) were characterized ex vivo by intracellular cytokine (IFN-γ and TNF) and surface marker (CD107a) staining. (**A**) Percentage of individuals with ex vivo CD4^+^ (left), CD8^+^ (middle), and both CD4^+^ and CD8^+^ (right) T cell responses to the SARS-CoV-2 spike–specific peptide pools. (**B**) Frequencies of spike-specific CD4^+^ T cells after complete vaccination assessed ex vivo. (**C**) Exemplary flow cytometry data of indicated cytokines and surface marker shown for CD4^+^ T cells for one donor after complete vaccination (BNT162b2 x BNT162b2) with an mRNA vaccine. (**D**) Proportion of samples with nonfunctional (0), monofunctional (1), bifunctional (2), or trifunctional (3) spike-specific CD4^+^ T cells after complete vaccination. (**E**) Frequencies of spike-specific CD8^+^ T cells after complete vaccination assessed ex vivo. (**F**) Exemplary flow cytometry data of indicated cytokines and surface marker shown for CD8^+^ T cells for one donor after complete vaccination (BNT162b2 x BNT162b2) with an mRNA vaccine. (**G**) Proportion of samples with nonfunctional (0), monofunctional (1), bifunctional (2), or trifunctional (3) spike-specific CD8^+^ T cells after complete vaccination. T cell responses were considered positive if the detected frequency of cytokine-positive CD4^+^ or CD8^+^ T cells was ≥3-fold higher than the frequency in the negative control and at least 0.1% of total CD4^+^ or CD8^+^ T cells. Responders are represented by colored symbols, and nonresponders are represented by clear symbols. Symbol shapes indicate the different vaccine products received by the donors. (A) Fisher’s exact test. (B and E) Box plots show the median with the 25th and 75th percentiles, and whiskers represent the minimum and maximum; Kruskal-Wallis test was used. If *P* values are not shown, then the results were not significant. FSC, forward scatter; FITC, fluorescein isothiocyanate.

Spike-specific IFN-γ T cell responses were observed ex vivo for 100% of mRNA- (*n* = 24) and heterologous-vaccinated donors (*n* = 15; Fig. 1C and Table 1). The cohort of vector-vaccinated donors (*n* = 9) showed a significantly reduced response rate (67%) compared with the other vaccination regimens (Fig. 1C). In COVID-19 convalescent donors (*n* = 16), spike-specific IFN-γ T cell responses were detected in 88% of the donors. A total of 16% of prepandemic donors never exposed to SARS-CoV-2 (Pre, *n* = 31; Fig. 1C) showed low-intensity cross-reactive spike-specific T cell responses (Fig. 1D). Intensity of spike-specific T cell responses did not significantly differ between the three vaccination cohorts and convalescent donors (Fig. 1D). However, mRNA- (median calculated spot counts, 71) and heterologous-vaccinated donors (median, 69) exhibited a two- to threefold increased T cell response intensity compared with vector-vaccinated (median, 24) and convalescent donors (median, 24; Fig. 1D). No correlation was observed between the time point of sample collection after complete vaccination (fig. S2A); demographic donor characteristics comprising body mass index (BMI), age, sex, or side effects after vaccination as well as clinical symptoms of COVID-19 (as assessed by questionnaires) of complete vaccination (Table 1 and fig. S2, B to E) and convalescent (Table 2 and fig. S2, F to H) individuals, respectively; and the intensity of spike-specific IFN-γ T cell responses.

**Table 2.** Characteristics of SARS-CoV-2 convalescent donors. Convalescents showed asymptomatic to mild COVID-19. By the time of sample collection, the wild-type SARS-CoV-2 was circulating, and VOCs emerged at a later time point. None of the donors were hospitalized or required oxygen treatment. Awareness of disease symptoms was assessed by a questionnaire.

	Convalescent donor cohort
**Number of donors**	16
**Age (years)**	
Median	46
Range	19–83
**Sex [*n* (%)]**	
Female	11 (68.8)
Male	5 (31.2)
**Comorbidities [*n* (%)]**	
High blood pressure	6 (37.5)
Cardiovascular disease	0 (0.0)
Blood sugar disorder	2 (12.5)
Chronic lung disease	0 (0.0)
Cancer disease	0 (0.0)
**Sample collection date**	July 2020
**Interval positive test to sample collection (weeks)**	
Median	17
Range	13–19
**Awareness of symptoms [*n* (%)]**	
No	3 (18.75)
Mild	2 (12.5)
Moderate	6 (37.5)
Severe	5 (31.25)
**Febrile illness (≥38.0°C)**	
No	10 (62.5)
Yes	6 (37.5)

After 12-day T cell expansion (fig. S3A), the percentage of donors with detectable spike-specific T cell responses was increased to 100% for all vaccinated groups and the convalescent cohort and to 97% for prepandemic donors (fig. S3B). Significantly increased intensity of IFN-γ T cell responses for vaccinated donors (median mRNA, 1286; vector, 1281; heterologous, 2602) and convalescent donors (median, 2946) was observed compared with prepandemic donors (median, 112; fig. S3C) and with ex vivo responses (fold change mRNA, 18; vector, 53; heterologous, 38; fig. S3D). This indicates potent expandability of vaccine-induced T cells upon SARS-CoV-2 exposure.

There are differences in SARS-CoV-2 T cell cross-reactivity to common cold human coronaviruses (HCoVs) of the N-terminal (less HCoV homologous) and C-terminal domain of the spike protein (*18*). To assess whether these differences affect vaccine-induced T cell responses, we performed IFN-γ ELISpot assays individually for the three different spike pools (Fig. 1E and fig. S3E). In the mRNA-vaccinated, heterologous-vaccinated, and convalescent cohort, the most frequently recognized peptide pool was the Prot_S1 pool, with 96, 100, and 75% of individuals showing an ex vivo response against this pool, respectively (Fig. 1E). In the vector-vaccinated cohort, the Prot_S+ pool was recognized by T cells from the majority of donors (67%, not reaching a level of significance compared with the other peptide pools; Fig. 1E). After 12-day T cell expansion, the differences in pool-specific recognition rates within the cohorts were upheld (fig. S3E). Most individuals vaccinated with mRNA (75%) or a heterologous scheme (80%) showed ex vivo T cell responses against all three spike pools, whereas only 33% of vector-vaccinated individuals recognized all pools (Fig. 1F). A total of 44% of convalescent donors exhibited T cell responses against all pools (Fig. 1F). After 12-day T cell expansion, at least 78% of vaccinated donors recognized all peptide pools independent of vaccination regimen (fig. S3F). For the prepandemic cohort, we detected no relevant differences in the recognition rate (up to 10% ex vivo and 66% after a 12-day expansion) and intensity of cross-reactive T cell responses for the three peptide pools (Fig. 1, E and F, and fig. S3, E and F).

T cell cross-recognition of the current dominant Omicron variant–specific mutations in the spike protein was assessed by ELISpot assays with spike-derived Omicron BA.1 and BA.2 variant-specific pools (Fig. 2A and table S2). Cross-recognition of the BA.1- and BA.2-mutated regions by vaccine-induced T cells was observed for the majority of mRNA-vaccinated (69 and 85% for BA.1 and 69 and 85% for BA.2) and heterologous-vaccinated (80 and 90% for BA.1 and 60 and 100% for BA.2) donors ex vivo and after 12-day T cell expansion, respectively. T cell cross-recognition of BA.1- and BA.2-mutated regions of the spike protein was reduced in the vector-vaccinated cohort, with 20 and 0% recognition ex vivo and 25 and 25% recognition after 12-day T cell expansion, respectively (Fig. 2, B to E, and fig. S4). In summary, our results showed induction of broad spike-specific T cell responses, particularly for mRNA- and heterologous-vaccinated individuals, that resembled the responses observed in convalescent donors.

### Characterization of SARS-CoV-2 spike–specific T cell responses after complete vaccination

Ex vivo intracellular cytokine and surface marker staining revealed vaccine-induced spike-specific CD4^+^ T cell responses for the majority of vaccinated donors of all regimens and in convalescent donors (86% for mRNA vaccinated, 71% for vector vaccinated, 70% for heterologous vaccinated, and 75% for convalescents). The percentages of donors with CD8^+^ (57% for mRNA vaccinated, 71% for vector vaccinated, 30% for heterologous vaccinated, and 42% for convalescents) as well as with both CD4^+^ and CD8^+^ T cell responses (57% for mRNA vaccinated, 57% for vector vaccinated, 30% for heterologous vaccinated, and 33% for convalescents) were generally lower compared with CD4^+^ T cell responses (Fig. 3A). The low frequency of cross-reactive T cell responses detected in the prepandemic cohort in the IFN-γ ELISpot assay was mediated by CD8^+^ T cells (0% CD4^+^ T cells and 17% CD8^+^ T cells; Fig. 3A). Vaccine-induced CD4^+^ T cells displayed a T helper 1 (T_H_1) phenotype, showing mainly positivity for tumor necrosis factor (TNF) and, to a lesser extent, for CD107a and IFN-γ/TNF, and were negative for the T_H_2 marker interleukin-4 (IL-4) comparably with SARS-CoV-2–specific T cells in convalescent donors (Fig. 3, B to D, and fig. S5A). A significantly increased frequency of TNF^+^CD4^+^ T cells was observed for the mRNA-vaccinated cohort compared with the vector- and heterologous-vaccinated groups and for TNF^+^IFN-γ^+^CD4^+^ T cells compared with the vector-vaccinated group. CD8^+^ T cell responses, in terms of frequencies of cytokine-producing cells and the ability to produce multiple cytokines, also showed a similar profile in vaccinated donors and convalescent individuals with particular positivity for IFN-γ. Prepandemic donors had lower frequencies of cytokine-producing cells, significantly reduced for IFN-γ compared with vector- and mRNA-vaccinated donors (Fig. 3, E to G). No significant differences could be observed between CD8^+^ T cell responses and functionality in individuals vaccinated with different vaccination regimens or in convalescent donors.

Comparable frequencies of vaccine-induced memory CD45RO^+^TNF^+^CD4^+^ T cells were observed in the mRNA and heterologous vaccine cohort and the convalescent cohort (86% for mRNA vaccinated, 70% for heterologous vaccinated, and 58% for convalescents), whereas no CD45RO^+^TNF^+^CD4^+^ T cells could be detected in donors vaccinated with a vector-based vaccine regimen (fig. S5B). Cytokine-positive CD8^+^ memory T cells (CD45RO^+^IFN-γ^+^CD8^+^ T cell) were observed to a much lower extent (21% for mRNA vaccinated, 43% for vector vaccinated, 0% for heterologous vaccinated, and 0% for convalescents; fig. S5C).

In conclusion, no significant differences could be observed between CD4^+^ and CD8^+^ T cell responses in individuals vaccinated with different vaccination regimens and in convalescent donors. However, the ability of vector-vaccinated donors to produce several cytokines was reduced compared with that of the other vaccination regimens.

### Effects of booster vaccination on spike-specific immune responses to mRNA and heterologous vaccination regimens

**Fig. 4.**
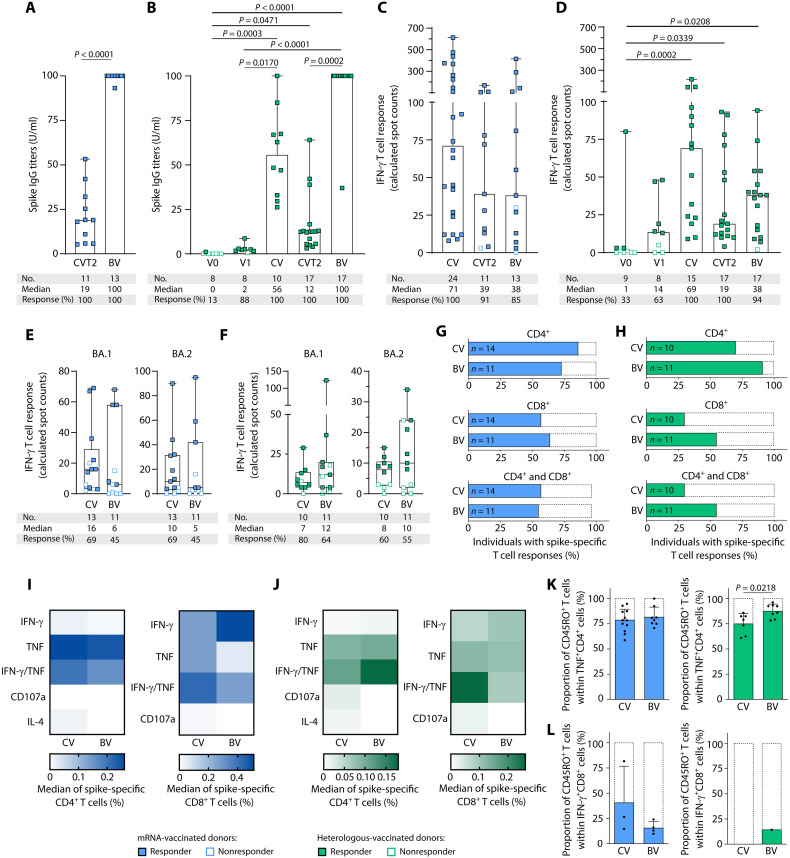
T cell and antibody responses of mRNA- and heterologous-vaccinated individuals after booster vaccination. (**A** to **D**) Time course of spike antibody titers (A and B) and intensities of ex vivo IFN-γ T cell responses in terms of calculated spot counts were assessed by IFN-γ ELISpot assays targeting spike-specific peptide pools (C and D) after mRNA (A and C) and heterologous (B and D) vaccination before (V0), 1 month after the first (V1) and complete vaccination (CV), 6 months after complete vaccination (CVT2), and 1 month after boost vaccination (BV). For results of paired samples from the same donors at each time point, please refer to fig. S9 (paired samples *n* = 8 for heterologous vaccination and *n* = 2 for mRNA vaccination). (**E** and **F**) Intensities of ex vivo IFN-γ T cell responses against the SARS-CoV-2 BA.1 and BA.2 mutation pools at complete vaccination and boost vaccination for mRNA- and heterologous-vaccinated donors, respectively. Responders are represented by colored symbols, and nonresponders are represented by clear symbols. (**G** to **L**) T cell responses were characterized by ex vivo intracellular cytokine and surface marker staining. T cell responses were considered positive if the detected frequency of cytokine-positive CD4^+^ or CD8^+^ T cells was ≥3-fold higher than the frequency in the negative control and minimum of 0.1% of total CD4^+^ or CD8^+^ T cells. (G and H) Percentage of individuals with CD4^+^ (top), CD8^+^ (middle), and both CD4^+^ and CD8^+^ (bottom) ex vivo T cell responses to spike-specific peptide pools during the course of mRNA (G) and heterologous (H) vaccination. (I and J) Heatmaps showing the percentages of cytokine- and surface marker–expressing CD4^+^ and CD8^+^ T cells ex vivo after complete and boost vaccination after mRNA (I) and heterologous (J) vaccination. (K) Proportion of TNF^+^CD4^+^ spike-specific T cells expressing the T cell memory marker CD45RO after complete vaccination (*n* = 12 and *n* = 7) and boost vaccination (*n* = 8 and *n* = 8) for mRNA- and heterologous-vaccinated donors, respectively. (L) Proportion of IFN-γ^+^CD8^+^ spike-specific T cells expressing the T cell memory marker CD45RO after complete (*n* = 3 and *n* = 0) and boost vaccination (*n* = 4 and *n* = 1) for mRNA- and heterologous-vaccinated donors, respectively. (A and B) Antibody titers are shown in units per milliliter (1 U/ml corresponds to 21.80 binding antibody units/ml). (A to D) Data are presented as scatter dot plots with the median, and whiskers show the maximum. (E and F) Box plots show the median with the 25th and 75th percentiles, and whiskers represent the minimum and maximum. (K and L) Data are presented as scatter dot plots with the mean, and error bars indicate SD. (A, K, and L) Mann-Whitney *U* test was used. (B to F) Kruskal-Wallis test was used. (G and H) Fisher’s exact test was used. If *P* values are not shown, then results were not significant.

Spike-specific antibody and T cell responses were assessed over time, at baseline before vaccination (V0), 1 month after the first (V1), after complete vaccination, 6 months after complete vaccination, and 1 month after the booster vaccination for mRNA- and heterologous-vaccinated donors. Booster vaccination induced a significant (up to eightfold) increase in spike-specific antibody levels, with immunoglobulin G (IgG) titers similarly enhanced from a median of 19 to 100 for mRNA-vaccinated individuals and from a median of 12 to 100 for heterologous-vaccinated donors compared with the time point 6 months after complete vaccination (Fig. 4, A and B, and fig. S6A). Spike-specific T cell responses were assessed by IFN-γ ELISpot assays ex vivo (Fig. 4, C and D) and after 12-day T cell expansion (fig. S6, C and D) for different time points after vaccination. Ex vivo IFN-γ T cell responses peaked comparably after complete vaccination for both vaccination regimens (median mRNA, 71; heterologous, 69), being about two- to threefold higher compared with 6 months after complete vaccination (median mRNA, 39; heterologous, 19; Fig. 4, C and D, and fig S6B). In contrast to antibody responses, the increase in T cell response intensity through boost vaccination, in terms of calculated spot counts, did not reach levels of significance, neither ex vivo nor after a 12-day T cell expansion (Fig. 4, A to D, and fig. S6, A to D). No correlations could be observed between IFN-γ T cell response intensity and BMI, age, sex, and donor-reported side effects after booster vaccination (fig. S2, I to L). The number of different spike-derived peptide pools that resulted in an ex vivo detectable T cell response (pool recognition rate) was highest after complete vaccination for both vaccination regimens and was not altered or increased by the booster vaccination. Spike-specific T cells showed potent expandability, resulting in T cell responses against all three spike peptide pools after 12-day T cell expansion at all time points after vaccination (figs. S6, C to F, and S7). Cross-recognition of the Omicron BA.1- and BA.2-mutated regions of the spike protein after booster vaccination in donors vaccinated with mRNA (45 and 91% for BA.1 and 45 and 91% for BA.2 donors with T cell response) or heterologous regimen (64 and 91% for BA.1 and 55 and 82% for BA.2 donors with T cell response) ex vivo and after 12-day T cell expansion, respectively, was comparable with the results after complete vaccination (Fig. 4, E and F, and fig. S6, G and H).

Comparison of vaccine-induced T cell phenotypes and functionality after complete vaccination and booster vaccination using ex vivo intracellular cytokine and surface marker staining showed no differences in the proportion of donors developing CD4^+^ and CD8^+^ T cell responses for the two vaccination cohorts (mRNA: CD4^+^ T cells 86% versus 73% and CD8^+^ T cells 57% versus 64%, respectively), with a nonsignificant increase in donors with vaccine-induced CD4^+^, CD8^+^, and CD4^+^ and CD8^+^ T cells after booster vaccination in the heterologous-vaccinated cohort (heterologous: CD4^+^ T cells 70% versus 91% and CD8^+^ T cells 30% versus 55%, respectively; Fig. 4, G and H, and fig. S8). Booster vaccination–induced CD4^+^ T cells in the mRNA and heterologous vaccine cohorts displayed a T_H_1 phenotype, showing mainly positivity for TNF and, to a lesser extent, for CD107a and IFN-γ/TNF, and were negative for the T_H_2 marker IL-4, comparable with the T cell responses observed after complete vaccination (fig. S9, A and B). CD8^+^ T cell responses, in terms of frequencies of cytokine-producing cells and the ability to produce multiple cytokines, also showed a similar profile in both vaccination cohorts after complete and booster vaccination with particular positivity for IFN-γ (Fig. 4, I and J, and fig. S8). Within the vaccine-induced TNF-producing spike-specific T cells, the proportion of CD45RO^+^CD4^+^ memory T cells showed a slight increase after booster vaccination compared with complete vaccination in both vaccination cohorts (mRNA: 78% versus 82%; heterologous: 75% versus 88%; Fig. 4K and fig. S9, C to F). For vaccine-induced IFN-γ–producing spike-specific CD8^+^ T cells, this increase in memory T cell response is only detected after heterologous vaccination (mRNA: 41% versus 16%; heterologous: 0% versus 14%; Fig. 4L).

In summary, the booster vaccination led to a significant increase in anti-spike IgG responses, which show a marked decline 6 months after complete vaccination. In contrast, anti-spike T cell responses remained stable over time after complete vaccination, with no significant effect of booster vaccination on the total intensity and frequency of T cell responses or on cross-recognition of Omicron BA.1 and BA.2 mutations within the spike protein.

## DISCUSSION

T cell immunity is central for the control of viral infections. Although the role of antiviral T cell response is extensively studied during acute SARS-CoV-2 infection and COVID-19 (*13*, *15*, *19*, *20*), the induction of T cells upon vaccination with the different approved COVID-19 vaccines is studied less extensively (*12*, *17*). This study reports on T cell immunity after complete and booster vaccination regimens in comparison with SARS-CoV-2 T cell responses in convalescents and prepandemic donors.

In line with previous reports (*21*, *22*), the frequency and intensity of spike-specific T cell responses were lower in vector-vaccinated donors compared with mRNA- and heterologous-vaccinated individuals, who showed comparable T cell responses. Of note, the observed difference between vaccination regimens vanished after in vitro T cell expansion, indicating potent expandability of vaccine-induced T cells upon virus encounter. Besides the expandability of virus-specific T cells (*23*), the diversity of T cell responses, i.e., recognition of multiple T cell epitopes, is shown to be central to combat viral disease, including SARS-CoV-2 (*15*, *24*). We showed that vaccine-induced T cells responded to different peptide pools covering the whole spike protein, indicating highly diverse T cell immunity by the different vaccination regimens. Our data on the expandability and broadness of vaccine-induced T cell responses indicated that mRNA, vector, and heterologous vaccination regimens can be recommended in the future to induce protective T cell immunity.

Comparison with spike-specific T cell responses induced in nonhospitalized convalescent individuals revealed similar frequency and intensity of T cells induced by different vaccination regimens. Of note, the phenotype and functionality of vaccine-induced CD4^+^ and CD8^+^ T cells also resembled those after natural infection (*25*). The induction of both CD4^+^ and CD8^+^ T cells has been shown to be central for effective T cell immunity in infectious and malignant diseases (*26*).

Cross-reactivity of T cells for different virus species or even among different pathogens is a well-known phenomenon postulated to enable heterologous immunity to a pathogen after exposure to a nonidentical pathogen (*27*). In SARS-CoV-2, cross-reactive T cells are associated with protection against infection in COVID-19 contacts (*28*) and with enhanced immune responses upon infection and vaccination (*18*). Here, we showed high frequencies of spike-specific T cell responses in a cohort of prepandemic, unexposed donors after in vitro T cell expansion. In line with previous reports (*18*, *20*), the intensity and diversity of these preexisting T cell responses were significantly lower than in convalescent and vaccinated individuals. In contrast to previous reports (*18*), we could also show cross-reactive T cell responses against the Prot_S1 peptide pool covering the complete N-terminal part of the S1 domain of the spike protein, which is described as less HCoV homologous than the C-terminal section covered by the Prot_S peptide pool, indicating that cross-reactivity is not only based on sequence similarity but also based on physiochemical and human leukocyte antigen (HLA)–binding properties (*29*, *30*).

Application of a booster vaccination after complete vaccination shows beneficial effects in terms of protection from SARS-CoV-2 infection and severe courses of COVID-19 (*31*, *32*). In line with previous reports, we showed a significant increase in IgG titers after booster vaccination for both mRNA and heterologous vaccination (*33*). In contrast, the frequency and intensity of T cell responses were not significantly boosted by the additional vaccination; however, T cell responses also did not exhibit such a marked decline after the complete vaccination compared with antibody responses. This is in line with reports after SARS-CoV-2 infection, showing a rapid antibody decline and the persistence of T cell immunity (*9*). No differences between mRNA and heterologous vaccination were observed in terms of T cell frequency, intensity, and ability of CD4^+^ T cells to produce multiple cytokines after booster vaccination. Of note, cytokine production in CD8^+^ T cells was only boosted in donors who received three doses of mRNA vaccine. These data indicated that boost vaccination is of particular relevance for the amelioration of antiviral antibody activity, whereas robust T cell immunity is already established after complete vaccination.

We further observed the cross-recognition of the Omicron BA.1- and BA.2-mutated regions of the spike protein by vaccine-induced T cells after complete and booster vaccination for most of the donors in the mRNA- and heterologous-vaccinated cohorts. This is in line with the cross-reactivity potential of SARS-CoV-2–specific T cells to HCoV (*18*, *28*) and provides the basis for the reported conservation of vaccine-induced T cell responses against different SARS-CoV-2 variants (*12*, *17*). This cross-reactivity is suggested to balance the lack of neutralizing antibodies targeting newly arising VOCs (*34*) and thus to prevent severe COVID-19 in vaccinees. These data on the cross-recognition potential of vaccine-induced T cells indicate that robust T cell immunity toward Omicron variants is also induced from complete vaccination.

There are several limitations to our study. We had a limited number of samples available, which particularly affected the vector-vaccinated group because vector-based vaccines stopped being recommended by German governments in mid-2021 (*35*). The other main limitation is the restricted number of paired samples for the analysis over time.

Together, our work shows that complete vaccination against COVID-19 induces broad spike-specific CD4^+^ and CD8^+^ T cell immunity by different vaccination regimens that resembles T cell responses after natural SARS-CoV-2 infection. Moreover, booster vaccination seems to be of particular relevance for the amelioration of antiviral antibody activity, because T cell responses are not markedly boosted by a third vaccination.

## MATERIALS AND METHODS

### Study design

This prospective cohort study was initiated in 2021 and describes T cell responses in donors vaccinated with different COVID-19 vaccines after complete and booster vaccination (regimens described in more detail below) compared with convalescent and prepandemic donors. Starting in January 2021, the German population was recommended to get vaccinated with the approved COVID-19 vaccines (BNT162b2, mRNA-1273, ChAdOx1, or Ad26.COV2.S), and volunteers were asked to participate in our study, which aimed to identify differences in T cell responses after the different vaccination regimens. T cell responses against the whole spike protein and against the Omicron BA.1 and BA.2 variant mutations were assessed. The control groups included samples collected from volunteer convalescents in 2020 after positive polymerase chain reaction (PCR) test and prepandemic samples collected before March 2017. No randomization was performed, and blinding was not appropriate for this study. The methods and assays used were standardized to prevent batch effects. Data for the time point before and after the first and complete vaccination of the same donor were obtained in the same assay, and data before and after booster vaccination were obtained in the same assay.

### Donors and blood samples

Peripheral blood mononuclear cells (PBMCs) from vaccinated donors, COVID-19 convalescents, and prepandemic healthy volunteers, collected between August 2015 and March 2017 at the University Hospital Tübingen and the Cancer Research Department Rhein-Main (Hospital Nordwest), were isolated by density gradient centrifugation and stored at −80°C for short-term storage or in liquid nitrogen until further use for subsequent T cell–based assays. Informed consent was obtained in accordance with the Declaration of Helsinki protocol. The study was performed according to the guidelines of the local ethics committees (179/2020/BO2, MC 288/2015, and 2021-2305-evBO).

### Donors vaccinated with different COVID-19 vaccination regimens

To assess spike-specific immune responses after vaccination, we collected blood samples from donors vaccinated with three different COVID-19 vaccine regimens. The mRNA-based vaccine cohort includes healthy volunteers vaccinated two (complete vaccination) or three times (booster vaccination) either with mRNA-1273 or with BNT162b2. The heterologous vaccination group received one dose of ChAdOx1 followed by one (complete vaccination) or two doses (booster vaccination) of either mRNA-1273 or BNT162b2. Donors of the vector-based vaccine group received either two doses of AZD1222 or one dose of Ad26.COV2.S for complete vaccination. Donor characteristics and side effects after vaccination of the cohorts (*n* = 61) are provided in Table 1 and were assessed by a questionnaire. Donors reporting headache, fever, or shivering after vaccination were classified as donors with side effects.

### SARS-CoV-2 convalescent individuals

To delineate differences of SARS-CoV-2 immune responses in vaccinated participants to immune responses after natural infection, we used a reference group of COVID-19 convalescent individuals, described previously (*20*), for comparison. SARS-CoV-2 infection was confirmed by real-time PCR after nasopharyngeal swab. Sample collection for human COVID-19 convalescents (*n* = 16) was performed in July 2020, 94 to 130 days (median, 117 days) after positive PCR. By the time of sample collection, the wild-type SARS-CoV-2 was circulating, and VOCs emerged at a later time point. Donor characteristics and COVID-19 symptoms were assessed by a questionnaire. Details are provided in Table 2. Written informed consent was obtained in accordance with the Declaration of Helsinki protocol (179/2020/BO2).

### IFN-γ ELISpot assay

ELISpot assays were performed ex vivo or after a 12-day in vitro expansion. For in vitro expansion, PBMCs were pulsed with overlapping 15-mer peptide pools covering the entire spike protein (Miltenyi, PepTivator SARS-CoV-2 Prot_S, PepTivator SARS-CoV-2 Prot_S+, and PepTivator SARS-CoV-2 Prot_S1; Fig. 1A) or the Omicron BA.1- and BA.2-mutated regions (Miltenyi, PepTivator SARS-CoV-2 Prot_S B.1.1.529/BA.1 Mutation Pool and PepTivator SARS-CoV-2 Prot_S B.1.1.529/BA.2 Mutation Pool) (0.02 nmol per peptide per milliliter) and cultured for 12 days, adding IL-2 (20 U/ml; Novartis) on days 3, 5, and 7. Peptide-stimulated (in vitro expanded) or freshly thawed (ex vivo) PBMCs were analyzed by IFN-γ ELISpot assay, as described previously (*20*). In brief, 100,000 to 300,000 cells per well were incubated in 96-well ELISpot plates coated with anti–IFN-γ antibody (2 μg/ml; clone 1-D1K, MabTech, catalog no. 3420-3-250, RRID: AB_907283) with peptide pools (0.01 nmol per peptide per milliliter). Phytohemagglutinin (Sigma-Aldrich) served as a positive control. An irrelevant HLA-DR–restricted control peptide (ETVITVDTKAAGKGK, FLNA_HUMAN1669-1683) in double-distilled water served as a negative control. After 24 hours of incubation, spots were revealed with anti–IFN-γ–biotinylated detection antibody (0.3 μg/ml; clone 7-B6-1, MabTech, catalog no. 3420-6-250, RRID: AB_907273), ExtrAvidin-Alkaline Phosphatase (1:1000 dilution; Sigma-Aldrich), and bromochloroindolyl phosphate/nitro blue tetrazolium (Sigma-Aldrich). Spots were counted using an ImmunoSpot S6 analyzer (CTL). T cell responses were considered positive, and donors as responders, if the mean spot count of the technical replicates normalized to 300,000 cells was at least three spots ex vivo and six spots after a 12-day in vitro expansion and threefold higher than the mean spot count of the negative control normalized to 300,000 cells (*15*, *36*). The intensity of T cell responses is depicted as calculated spot counts, which represent the sum of mean spot count normalized to 300,000 cells for all three tested spike-specific peptide pools subtracting the normalized mean spot count of the respective negative control.

### Intracellular cytokine and cell surface marker staining

Peptide-specific T cells were characterized by cell surface marker and intracellular cytokine staining (ICS) as previously described (*20*). In brief, 250,000 to 1,000,000 PBMCs were incubated over 12 to 14 hours with the 15-mer peptide pools covering the entire spike protein or the negative control peptide, brefeldin A (Sigma-Aldrich), and GolgiStop (BD Biosciences). Phorbol 12-myristate 13-acetate and ionomycin (Sigma-Aldrich, catalog no. L1668) served as a positive control, and for the ex vivo ICS, staphylococcal enterotoxin B (SEB) (Sigma-Aldrich, catalog no. S4881) was used as an additional positive control. Staining was performed using Cytofix/Cytoperm solution (BD), Zombie Aqua (for ex vivo samples, 1:200 dilution; BioLegend), allophycocyanin (APC)/Cy7 anti-human CD4 (1:100 dilution; BioLegend, catalog no. 300518, RRID: AB_314086), phycoerythrin (PE)/Cy7 anti-human CD8 (1:400 dilution; Beckman Coulter, catalog no. 737661, RRID: AB_1575980), Pacific Blue anti-human TNF (1:120 dilution; BioLegend, catalog no. 502920, RRID: AB_528965), fluorescein isothiocyanate anti-human CD107a (1:100 dilution; BioLegend, catalog no. 328606, RRID: AB_1186036), PE anti-human IFN-γ monoclonal antibodies (1:200 dilution; BioLegend, catalog no. 506507, RRID: AB_315440), APC anti-human CD45RO (1:100 dilution; BioLegend, catalog no. 304210, RRID: AB_314426), and PE-Dazzle 594 anti-human IL-4 (1:25 dilution; BioLegend, catalog no. 500832, RRID: AB_2564036). Ex vivo samples were analyzed on a FACS LSRFortessa (BD; gating strategy; fig. S10). In this study, a T_H_1 response was defined as cells producing IFN-γ and TNF, and a T_H_2 response was defined as cells producing IL-4. T cell responses were considered positive if the detected frequency of cytokine-positive CD4^+^ or CD8^+^ T cells was ≥3-fold higher than the frequency in the negative control and minimum of 0.1%. The frequency of cytokine-positive cells was corrected for background by subtraction of the respective negative control values. Negative values were set to zero.

### SARS-CoV-2 anti-spike and anti-nucleocapsid antibody testing

The Siemens SARS-CoV-2 IgG (SCOVG) assay was performed on an automated ADVIA Centaur XPT System (Siemens Healthineers) according to the manufacturer’s instructions. The immunoassay detects anti-SCOVG antibodies directed against the S1 domain of the viral spike protein (including the immunologically relevant receptor binding domain). The Elecsys assay from Roche detecting high-affinity antibodies (including IgG) directed against the nucleocapsid protein of SARS-CoV-2 was performed according to the manufacturer’s instructions for samples collected at the University Hospital Tübingen. Results are reported in index values for the Roche assay and the SCOVG assay. For the latter, an index value of 1 corresponds to 1 U/ml; 1 U/ml can be converted to 21.80 binding antibody units/ml according to the manufacturer. The final interpretation of positivity is determined by an antibody titer of ≥1.0 U/ml given by the manufacturer. Values of <0.1 were set to 0.1. One hundred was the highest measurable index value with the SCOVG assay. Quality control was performed following the manufacturer’s instructions on each day of testing.

### Software and statistical analysis

Flow cytometric data were analyzed using FlowJo 10.7.1 (BD). Graphs were plotted using Inkscape 1.1 and GraphPad Prism 9.2.0. Statistical analyses were conducted using GraphPad Prism 9.2.0. Data are displayed as means ± SD, and box plots are displayed as median with 25 or 75% quantiles and minimum/maximum whiskers. Continuous data were tested for distribution, and individual groups were tested by the use of two-sided Fisher’s exact test, unpaired *t* test, unpaired Mann-Whitney *U* test, Kruskal-Wallis test, or paired Wilcoxon signed-rank test and Friedman test, all performed as two-sided tests. Correlation was tested using the Spearman test and linear regression. *P* values of <0.05 were considered statistically significant.

## Supplementary Material

20221101-1

## Notes

View/request a protocol for this paper from *Bio-protocol*.
