# Supplementary material for: Durable spike-specific T cell responses after different COVID-19 vaccination regimens are not further enhanced by booster vaccination
Source: Sci Immunol. 2022 Dec 16;7(78):eadd3899. doi: 10.1126/sciimmunol.add3899 (PMC9798886; doi:10.1126/sciimmunol.add3899)
Supplement: 20221101-1 [file sciimmunol.add3899.v1.pdf]

Science Immunology | REPORT | FIRST RELEASE

CORONAVIRUS

Durable spike-specific T-cell responses after different COVID-19 vaccination regimens are not further enhanced by booster vaccination

Copyright © 2022 The Authors, some rights reserved; exclusive licensee American Association for the Advancement of Science. No claim to original U.S. Government Works. Distributed under a Creative Commons Attribution License 4.0 (CC BY).

Yacine Maringer[1,2,3], Annika Nelde[1,2,3], Sarah M. Schroeder[1,2,4], Juliane Schuhmacher[1,3], Sebastian Hörber[5], Andreas Peter[5], Julia Karbach[6], Elke Jäger[6], Juliane S. Walz[1,2,3,7]*

Several COVID-19 vaccines are approved to prevent severe disease outcome following SARS-CoV-2 infection. Whereas the induction and functionality of anti-viral antibody response is largely studied, the induction of T cells upon vaccination with the different approved COVID-19 vaccines is less studied. Here, we reported on T-cell immunity four weeks and six months after different vaccination regimens and four weeks after an additional booster vaccination, in comparison to SARS-CoV-2 T-cell responses in convalescents and prepandemic donors using interferon-gamma ELISpot assays and flow cytometry. Increased T-cell responses and cross-recognition of B.1.1.529 Omicron variant-specific mutations were observed *ex vivo* in mRNA- and heterologous-vaccinated donors compared to vector-vaccinated donors. Nevertheless, potent expandability of T cells targeting the spike protein was observed for all vaccination regimens with frequency, diversity and the ability to produce several cytokines of vaccine-induced T-cell responses comparable to those in convalescent donors. T-cell responses for all vaccinated donors significantly exceeded preexisting cross-reactive T-cell responses in prepandemic donors. Booster vaccination led to a significant increase of anti-spike IgG responses, which showed a marked decline 6 month after complete vaccination. In contrast, T-cell responses remained stable over time following complete vaccination with no significant effect of booster vaccination on T-cell responses and cross-recognition of Omicron BA.1 and BA.2 mutations.This suggested that booster vaccination is of particular relevance for the amelioration of antibody response. Together, our work shows that different vaccination regimens induce broad and long-lasting spike-specific CD4$^+$ and CD8$^+$ T-cell immunity to SARS-CoV-2.

INTRODUCTION

During the Coronavirus Disease 2019 (COVID-19) pandemic, caused by the severe acute respiratory syndrome coronavirus 2 (SARS-CoV-2), several vaccines have been successfully developed, reducing transmission and preventing billions of people from severe disease outcome (*1*–*4*). Among the currently approved COVID-19 vaccines, the ChAdOx1 nCoV-19 adenovirus-based vector vaccine ChAdOx1, the human adenovirus type 26 (Ad26)-based vector vaccine Ad26.COV2.S and the two messenger ribonucleic acid (mRNA) vaccines BNT162b2 and mRNA-1273 are the most widely used in Europe and North America (*5*–*7*). Vaccination schedules comprise two doses of ChAdOx1, BNT162b2 and mRNA-1273 and one dose of Ad26.COV2.S for complete vaccination status (*1*–*4*). After reports of thromboembolic events following ChAdOx1 vaccination (*8*), several European governments recommended to complete vaccination with an mRNA vaccine after the first dose of ChAdOx1 (heterologous vaccination). To overcome waning vaccine immunity over time (*9*), the administration of an additional booster vaccine dose was approved in many countries 3–6 month after completion of vaccination (*10*).

COVID-19 vaccination induces both humoral immunity, mediated by B cell-derived antibodies, and cellular immunity, mediated by T cells (*2*). Although it is undisputed that neutralizing antibodies provide the first line of anti-viral defense (*11*, *12*), T-cell immunity is crucial to combat acute SARS-CoV-2 infection and for the development of long-term immunity (*13*). Whereas, antibody titers tend to wane fast and show limited neutralizing activity to newly arising variants of concerns (VOCs), T-cell memory is largely conserved against VOCs after prior SARS-CoV-2 infection (*14*, *15*) .

So far, research on SARS-CoV-2 vaccine-induced immunity is largely focused on anti-spike antibody titers and their ability to neutralize virus particles (*16*). Spike-specific T-cell responses induced upon different vaccination regimens are studied to a lesser extent, with first reports showing induction of both CD4$^+$ and CD8$^+$ T-cell responses after complete vaccination with different vaccination regimens. Moreover, T-cell responses are shown to be largely conserved against different SARS-CoV-2 variants including early B.1.1.529 (Omicron) variants, now dominant globally (*12*, *17*).

In this work, we provided an analysis of spike-specific T-cell responses and their cross-recognition of B.1.1.529 Omicron BA.1 and BA.2 variant-specific mutations after complete vaccination with mRNA, vector and heterologous vaccine regimens in comparison to COVID-19 convalescents and prepandemic donors. Additionally, we provided insight on the effects of a third mRNA booster vaccination after homologous and heterologous vaccination regimens on T-cell and antibody immunity.

[1]Department of Peptide-based Immunotherapy, University and University Hospital Tübingen, Tübingen, Germany. [2]Institute for Cell Biology, Department of Immunology, University of Tübingen, Tübingen, Germany. [3]Cluster of Excellence iFIT (EXC2180) "Image-Guided and Functionally Instructed Tumor Therapies", University of Tübingen, Tübingen, Germany. [4]Department of Otorhinolaryngology, Head and Neck Surgery, University Hospital Tübingen, Tübingen, Germany. [5]Institute for Clinical Chemistry and Pathobiochemistry, Department for Diagnostic Laboratory Medicine, University Hospital Tübingen, Tübingen, Germany. [6]Department of Oncology and Hematology, Krankenhaus Nordwest, Frankfurt, Germany. [7]Clinical Collaboration Unit Translational Immunology, German Cancer Consortium (DKTK), Department of Internal Medicine, University Hospital Tübingen, Tübingen, Germany.
*Corresponding author. Email: juliane.walz@med.uni-tuebingen.de

RESULTS

SARS-CoV-2 spike-specific T-cell responses after complete vaccination with different vaccination regimens

To assess spike-specific T-cell responses after complete vaccination (two doses of either BNT162b2, mRNA-1273 or ChAdOx1, one dose of Ad26.COV2.S, or one dose of vector vaccine ChAdOx1 followed by one dose of an mRNA vaccine for heterologous vaccine regimens), interferon-gamma (IFN-γ) enzyme-linked immunospot (ELISpot) assays were performed three to twelve weeks (median four weeks) after the complete vaccination dose (Table 1). Results were obtained using three different peptide pools covering various parts of the spike protein, with Prot_S1 covering the complete N-terminal S1 domain; Prot_S+ covering part of the C-terminal S2 domain and Prot_S comprising selected immunodominant sequence domains (Fig. 1A,B). Asymptomatic infections during the study period were excluded by testing for nucleocapsid antibodies (Fig. S1).

Spike-specific IFN-γ T-cell responses were observed *ex vivo* for 100% of mRNA- (n = 24) and heterologous-vaccinated donors (n = 15, Fig. 1C, Table 1). The cohort of vector-vaccinated donors (n = 9) showed a significantly reduced response rate compared (67%) to the other vaccination regimens (Fig. 1C). In COVID-19 convalescent donors (n = 16) spike-specific IFN-γ T-cell responses were detected in 88% of the donors. 16% of prepandemic donors never exposed to SARS-CoV-2 (Pre, n = 31, Fig. 1C) showed low-intensity cross-reactive spike-specific T-cell responses (Fig. 1D). Intensity of spike-specific T-cell responses did not significantly differ between the three vaccination cohorts and convalescent donors (Fig. 1D). However, mRNA- (median calculated spot counts 71) and heterologous-vaccinated donors (median 69) exhibited a two to three-fold increased T-cell response intensity compared to vector-vaccinated (median 24) and convalescent donors (median 24, Fig. 1D). No correlation was observed between the time point of sample collection after complete vaccination (Fig. S2A), demographic donor characteristics comprising body mass index (BMI), age, sex or side effects after vaccination as well as clinical symptoms of COVID-19 (as assessed by questionnaires) of complete vaccination (Fig. S2B-E, Table 1) and convalescent (Fig. S2F-H, Table 2) individuals, respectively, and the intensity of spike-specific IFN-γ T-cell responses.

After 12-day T-cell expansion (Fig. S3A), the percentage of donors with detectable spike-specific T-cell responses was increased to 100% for all vaccinated groups and the convalescent cohort and to 97% for prepandemic donors (Fig. S3B). Significantly increased intensity of IFN-γ T-cell responses for vaccinated donors (median mRNA 1,286; vector 1,281; heterologous 2,602) and convalescent donors (median 2,946) was observed compared to prepandemic donors (median 112, Fig. S3C) and to *ex vivo* responses (fold change mRNA 18, vector 53, heterologous 38, Fig. S3D). This indicates potent expandability of vaccine-induced T cells upon SARS-CoV-2 exposure.

There are differences in SARS-CoV-2 T-cell cross-reactivity to common cold human coronaviruses (HCoV) of the N- (less HCoV homologous) and C-terminal domain of the spike protein (*18*). To assess if these differences impact vaccine-induced T-cell responses, IFN-γ ELISpot assays were performed individually for the three different spike pools (Fig. 1E, Fig. S3E). In the mRNA-vaccinated, heterologous-vaccinated, and convalescent cohort the most frequently recognized peptide pool was the Prot_S1 pool, with 96%, 100% and 75% of individuals showing an *ex vivo* response against this pool, respectively (Fig. 1E). In the vector-vaccinated cohort the Prot_S+ pool was recognized by T cells from the majority of donors (67%, not reaching level of significance compared to the other peptide pools, Fig. 1E). After 12-day T-cell expansion, the differences in pool-specific recognition rates within the cohorts were upheld (Fig. S3E). Most individuals vaccinated with mRNA (75%) or a heterologous scheme (80%) showed *ex vivo* T-cell responses against all three spike pools, whereas only 33% of vector-vaccinated individuals recognized all pools (Fig. 1F). 44% of convalescent donors exhibited T-cell responses against all pools (Fig. 1F). After 12-day T-cell expansion at least 78% of vaccinated donorsrecognized all peptide pools independent of vaccination regimen (Fig. S3F). For the prepandemic cohort, we detected no relevant differences in recognition rate (up to 10% *ex vivo*, 66% after 12-day expansion) and intensity of cross-reactive T-cell responses for the three peptide pools (Fig. 1E,F, Fig. S3E,F).

T-cell cross-recognition of the current dominant Omicron variant-specific mutations in the spike protein, was assessed by ELISpot assays with spike-derived Omicron BA.1 and BA.2 variant-specific pools (Fig. 2A, Table S2). Cross-recognition of the BA.1 and BA.2 mutated regions by vaccine-induced T cells was observed for the majority of mRNA- (BA.1 69% and 85%, BA.2 69% and 85%) and heterologous- (BA.1 80% and 90%, BA.2 60% and 100%) vaccinated donors *ex vivo* and after 12-day T-cell expansion, respectively. T-cell cross-recognition of BA.1 and BA.2 mutated regions of the spike protein was reduced in the vector-vaccinated cohort with 20% and 0% recognition *ex vivo* and 25% and 25% recognition after 12-day T-cell expansion, respectively (Fig. 2B-E; Fig. S4). In summary, our results showed induction of broad spike-specific T-cell responses, in particular for mRNA- and heterologous-vaccinated individuals that resembled the responses observed in convalescent donors.

Characterization of SARS-CoV-2 spike-specific T-cell responses after complete vaccination

Ex vivo intracellular cytokine and surface marker staining revealed vaccine-induced spike-specific $CD4^+$ T-cell responses for the majority of vaccinated donors of all regimens and in convalescent donors (mRNA-, 86%; vector-, 71%; heterologous-vaccinated, 70%; convalescents, 75%). The percentages of donors with $CD8^+$ (mRNA-, 57%; vector-, 71%; heterologous-vaccinated, 30%; convalescents, 42%) as well as with both $CD4^+$ and $CD8^+$ T-cell responses (mRNA-, 57%; vector-, 57%; heterologous-vaccinated, 30%; convalescents, 33%) were generally lower compared to $CD4^+$ T-cell responses (Fig. 3A). The low frequency of cross-reactive T-cell responses detected in the prepandemic cohort in the IFN-γ ELISpot assay were mediated by $CD8^+$ T cells (0% $CD4^+$ T cells, 17% $CD8^+$ T cells, Fig. 3A). Vaccine-induced $CD4^+$ T cells displayed a T helper 1 (T_H1) phenotype, showing mainly positivity for tumor necrosis factor (TNF) and to a lower extent for CD107a and IFN-γ/TNF, and were negative for the T helper 2 (T_H2) marker IL-4 comparable to SARS-CoV-2-specific T cells in convalescent donors (Fig. 3B-D, Fig. S5A). A significantly increased frequency of TNF^+CD4^+ T cells was observed for the mRNA-vaccinated cohort compared to the vector- and heterologous-vaccinated groups, and for $TNF^+IFN\text{-}\gamma^+CD4^+$ T cells compared to the vector-vaccinated group. $CD8^+$ T-cell responses, in terms of frequencies of cytokine

Table 1. Characteristics of healthy volunteer cohorts after heterologous-, mRNA- or vector-based vaccination.

	mRNA vaccine cohort	vector vaccine cohort	heterologous vaccination cohort
**Number of donors**	35	10	17
**Age [years]**			
Median	38	n.a.	24 - 31
Range	25 -71	n.a	52
**Sex [n (%)]**			
Female	19 (54.3)	n.a.	11 (64.7)
Male	16 (45.7)	n.a.	6 (35.3)
**Comorbidities [n (%)]**			
High blood pressure	3 (12.5)	n.a.	1 (5.9)
Cardiovascular disease	1 (4.2)	n.a.	1 (5.9)
Blood sugar disorder	2 (8.3)	n.a.	0 (0.0)
Chronic lung disease	0 (0.0)	n.a.	1 (5.9)
Cancer disease	1 (4.2)	n.a.	0 (0.0)
n.a.	11	n.a.	0
**Vaccination schemes (CV)**			
BNT162b2 x BNT162b2	20 (83.3)		
mRNA-1273 × mRNA-1273	4 (16.7)		
ChadOx1 x ChadOx1		6 (60.0)	
Ad26.COV2.S		4 (40.0)	
ChadOx1 x BNT16b2			7 (46.7)
ChadOx1 × mRNA-1273			8 (53.3)
**Time points**			
**pre vaccination**			
Donors	-	-	9
**post first vaccination**			
Donors	-	-	8
Weeks post vaccination			
Median	-	-	10
Range	-	-	9 -10
**post complete vaccination**			
Donors	24	10	15
Weeks post vaccination			
Median	3	4	6
Range	3 – 10	3 – 8	3 – 12
Awareness of side effects [n (%)]			
Yes	9 (69.2)	n.a.	5 (50.0)
No	4 (30.8)	n.a.	5 (50.0)
n.a.	11	n.a.	5
**six months after complete vaccination**			
Donors	11	-	17
Weeks post vaccination			
Median	26	-	26
Range	21 -32	-	24 - 28
**post booster vaccination**			
Donors	13	-	17
Weeks post vaccination			
Median	4	-	4
Range	2 – 7	-	3 – 6
Awareness of side effects [n (%)]			
Yes	2 (18.2)	-	6 (35.3)
No	9 (81.8)	-	11 (64.7)
n.a.	2	-	0

continued on next page

The messenger ribonucleic acid (mRNA)-based vaccine cohort includes healthy volunteers vaccinated two (complete vaccination) to three times (booster vaccination) either with mRNA-1273 or with BNT162b2. Donors of the vector-based vaccine either received two doses of ChadOx1 (complete vaccination) or one dose of Ad26.COV2.S (complete vaccination). The heterologous vaccination group received one dose of ChadOx1 followed by one (complete vaccination) or two doses (booster vaccination) of either mRNA-1273 or BNT162b2. For mRNA- and heterologous-vaccinated donors, we analyzed two different time points after complete vaccination, time point of complete vaccination 3–12 weeks after complete vaccination, and time point six months after complete vaccination (21–32 weeks after complete vaccination). Figures 1 to 3 show T-cell responses after complete vaccination, analysis over different time points are shown in Figure 4. Figure 4 includes the identical results for the time point complete vaccination for mRNA- and heterologous-vaccinated donors as shown in figures 1 to 3. Experiments were not always conducted with all donors, depending on available cell numbers. n, number; V, vaccination;.

producing cells and the ability to produce multiple cytokines, also showed a similar profile in vaccinated donors and convalescent individuals with in particular positivity for IFN-γ. Prepandemic donors had lower frequencies of cytokine producing cells, significantly reduced for IFN-γ compared to vector and mRNA vaccinated donors (Fig. 3E-G). No significant differences could be observed between $CD8^+$ T-cell responses and functionality in individuals vaccinated with different vaccination regimens or in convalescent donors.

Comparable frequencies of vaccine-induced memory $CD45RO^{+-}$ TNF^+CD4^+ T cells were observed in the mRNA and heterologous vaccine cohort, and the convalescent cohort (mRNA-, 86%;

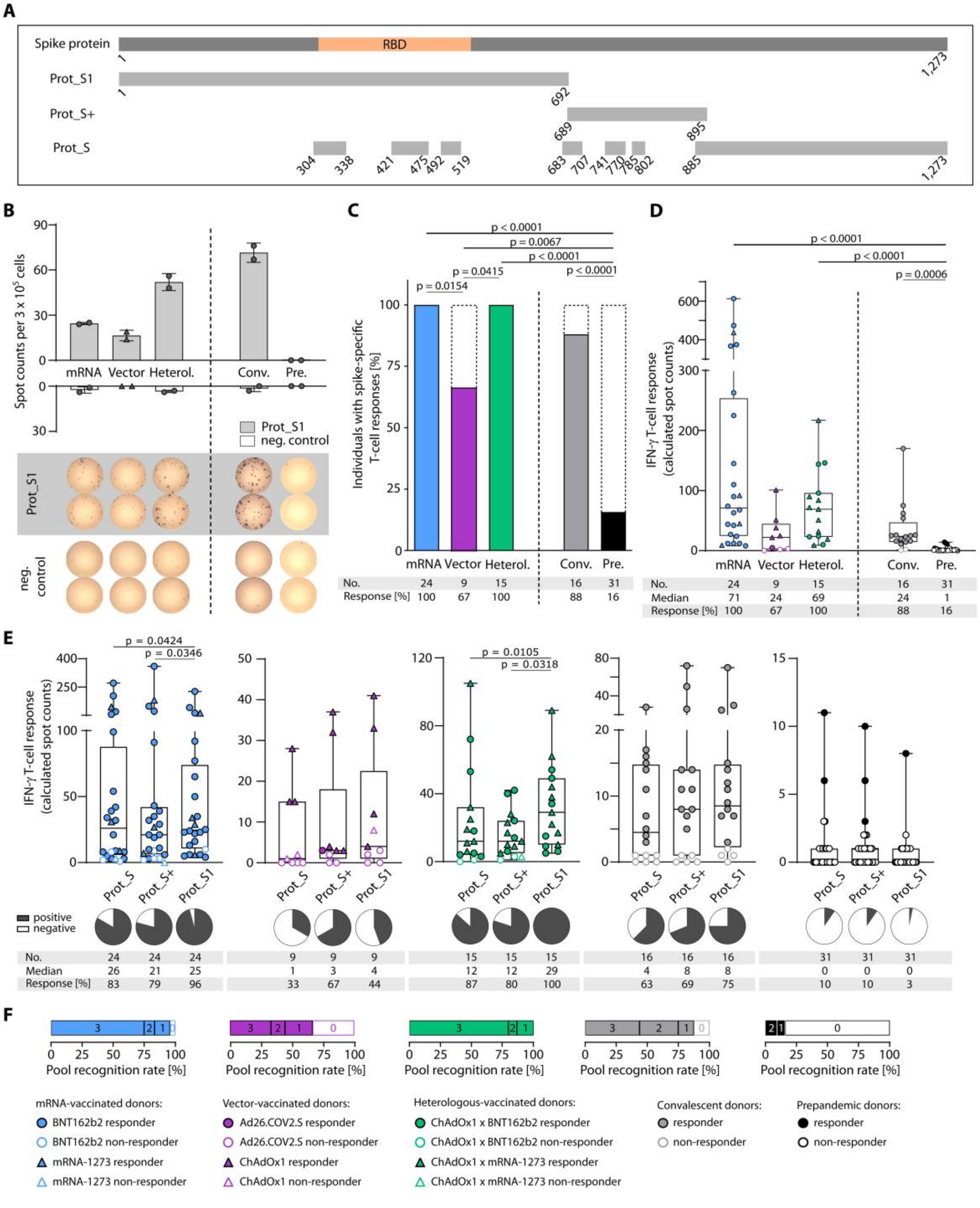

Fig. 1. *Ex vivo* immune responses to SARS-CoV-2 spike protein peptide pools following complete vaccination. A, Schematic depiction of the SARS-CoV-2 spike protein, and protein section coverage by the peptide pools (Prot_S1, Prot_S+, Prot_S) used for immunogenicity testing. **B-F,** *Ex vivo* T-cell responses after complete vaccination (CV, two doses of either BNT162b2, mRNA-1273 or ChAdOx1, one dose of Ad26.COV2.S, or one dose of the vector vaccine ChAdOx1 followed by one dose of an mRNA vaccine for heterologous (heterol.) vaccine regimens), compared to COVID-19 convalescents (Conv.) and prepandemic (Pre.) donors were assessed by interferon-gamma (IFN-γ) enzyme-linked immunospot (ELISpot) assays three to twelve weeks (median four weeks) after the last vaccine dose (sample collection post CV, Table 1). **B,** Representative example of *ex vivo* IFN-γ T-cell responses to the Prot_S1 peptide pool compared to a negative (neg.) control peptide, showing duplicates for one donor of each cohort. **C,D,** Percentage of individuals with *ex vivo* IFN-γ ELISpot T-cell responses (**C**), and intensities of IFN-γ T-cell responses in terms of calculated spot counts against the spike-specific peptide pools (**D**) after mRNA, vector or heterologous vaccination, compared to convalescents and prepandemic donors (summarized responses against the three spike-specific peptide pools). **E,** Intensities of *ex vivo* IFN-γ T-cell responses shown for the distinct spike protein peptide pools. **F,** Proportion of individuals (cohorts as indicated by color code) with responses to all three, two, one or none of the spike peptide pools. Responders are represented by colored symbols, non-responders by clear symbols. Symbol shapes indicate the different vaccine products received by the donors. **D,E,** box plots represent the median with 25th and 75th percentiles with minimum and maximum whiskers. **C,** Fisher's exact test was used. **D**, Kruskal-Wallis test was used, **E,** Friedman test was used, if no p values are shown results were not significant. RBD, receptor-binding domain; No., number.

Table 2. Characteristics of SARS-CoV-2 convalescent donors.

	Convalescent donor cohort
**Number of donors**	16
**Age [years]**	
Median	46
Range	19 - 83
**Sex [n (%)]**	
Female	11 (68.8)
Male	5 (31.2)
**Comorbidities [n (%)]**	
High blood pressure	6 (37.5)
Cardiovascular disease	0 (0.0)
Blood sugar disorder	2 (12.5)
Chronic lung disease	0 (0.0)
Cancer disease	0 (0.0)
**Sample collection date**	July 2020
**Interval positive test to sample collection (weeks)**	
Median	17
Range	13 -19
**Awareness of symptoms [n (%)]**	
No	3 (18.75)
Mild	2 (12.5)
Moderate	6 (37.5)
Severe	5 (31.25)
**Febrile illness (≥ 38.0°C)**	
No	10 (62.5)
Yes	6 (37.5)

Convalescents showed asymptomatic to mild COVID-19. By the time of sample collection the wildtype SARS-CoV-2 was circulating, variants of concern emerged at a later time point. None of the donors were hospitalized or required oxygen treatment. Awareness of disease symptoms was assessed by questionnaire. n, number.

heterologous-vaccinated, 70%; convalescents, 58%), whereas no $CD45RO^+TNF^+CD4^+$ T cell could be detected in donors vaccinated with a vector-based vaccine regimen (Fig. S5B). Cytokine-positive $CD8^+$ memory T cells ($CD45RO^+IFN$-γ^+CD8^+ T cell) were observed to a much lower extent (mRNA-, 21%; vector-, 43%; heterologous-vaccinated, 0%; convalescents, 0%, Fig S5C).

In conclusion, no significant differences could be observed between $CD4^+$ and $CD8^+$ T-cell responses in individuals vaccinated with different vaccination regimens and in convalescent donors. However the ability of vector-vaccinated donors to produce several cytokines was reduced compared to the other vaccination regimens.

Effects of booster vaccination on spike-specific immune responses to mRNA and heterologous vaccination regimens

Spike-specific antibody and T-cell responses were assessed over time, at baseline before vaccination (V0), one month after first (V1), after complete vaccination, six months after complete vaccination and one month after the booster vaccination for mRNA- and heterologous-vaccinated donors. Booster vaccination induced a significant up to 8-fold increase in spike-specific antibody levels, with IgG titers similarly enhanced from median 19 to 100 for mRNA-vaccinated individuals and from median 12 to 100 for heterologous-vaccinated donors compared to the time point six months after complete vaccination (Fig. 4A,B; Fig. S6A). Spike-specific T-cell responses were assessed by IFN-γ ELISpot assays *ex vivo* (Fig. 4C,D) and after 12-day T-cell expansion (Fig. S6C,D) for different time points after vaccination. *Ex vivo* IFN-γ T-cell responses peaked comparably after complete vaccination for both vaccination regimens (median mRNA, 71; heterologous, 69), being approximately two to three-fold higher compared to six months after complete vaccination (median mRNA, 39; heterologous, 19; Fig. 4C,D; Fig S6B). In contrast to antibody responses, the increase of T-cell response intensity through boost vaccination, in terms of calculated spot counts, did not reach levels of significance, neither *ex vivo* nor after 12-day T-cell expansion (Fig. 4A-D; Fig. S6A-D). No correlations could be observed between IFN-γ T-cell response intensity with BMI, age, sex and donor-reported side effects after booster vaccination (Fig. S2I-L). The number of different spike-derived peptide pools that resulted in an *ex vivo* detectable T-cell response (pool recognition rate) was highest after complete vaccination for both vaccination regimens and was not altered or increased by the booster vaccination. Spike-specific T cells showed potent expandability, resulting in T-cell responses against all three spike peptide pools after 12-day T-cell expansion at all time points after vaccination (Fig. S6C-F, Fig. S7). Cross-recognition of the Omicron BA.1 and BA.2 mutated regions of the spike protein after booster vaccination in donors vaccinated with mRNA (BA.1 45% and 91%, BA.2 45% and 91% donors with T-cell response) or heterologous regimen (BA.1 64% and 91%, BA.2 55% and 82% donors with T-cell response) *ex vivo* and after 12-day T-cell expansion, respectively was comparable to the results after complete vaccination (Fig. 4E,F; Fig. S6G,H).

Comparison of vaccine-induced T-cell phenotypes and functionality after complete vaccination and booster vaccination using *ex vivo* intracellular cytokine and surface marker staining showed no differences in the proportion of donors developing $CD4^+$ and $CD8^+$ T-cell responses for the two vaccination cohorts (mRNA: $CD4^+$ T cells 86% vs. 73%, $CD8^+$ T cells 57% vs. 64%, respectively) with a non-significant increase of donors with vaccine-induced $CD4^+$, $CD8^+$ as well as $CD4^+$ and $CD8^+$ T cells after booster vaccination in the heterologous-vaccinated cohort (heterologous: $CD4^+$ T cells 70% vs. 91%, $CD8^+$ T cells 30% vs. 55%, respectively, Fig. 4G,H; Fig. S8). Booster vaccination-induced $CD4^+$ T cells in the mRNA and heterologous vaccine cohorts displayed a T helper 1 (T_H1) phenotype, showing mainly positivity for TNF and to a lower extent for CD107a and IFN-γ/TNF and were negative for the T helper 2 (T_H2) marker IL-4, comparable to the T-cell responses observed after complete vaccination (Fig. S9A,B). $CD8^+$ T-cell responses, in terms of frequencies of cytokine-producing cells and the ability to produce multiple cytokines, also showed a similar

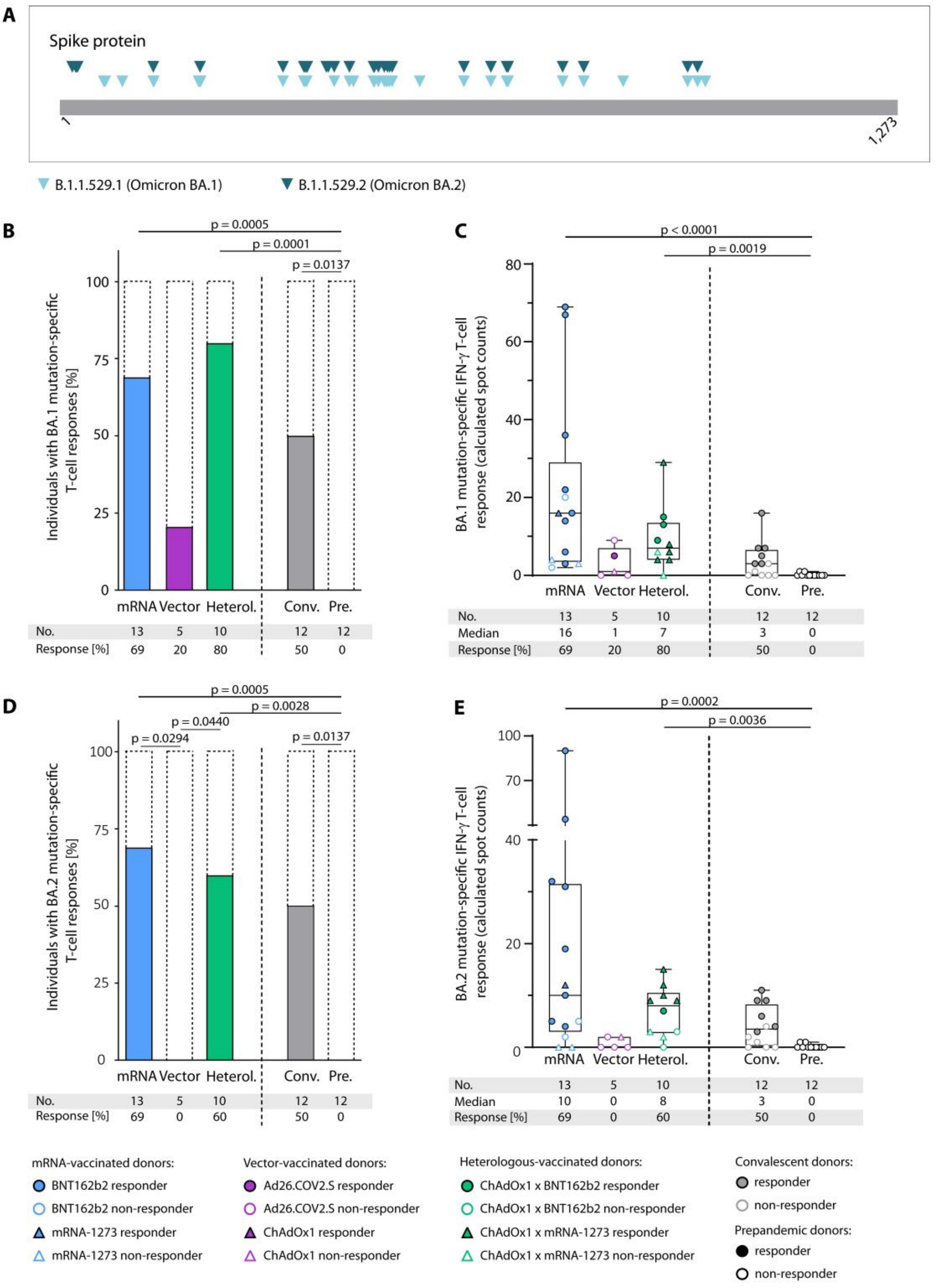

Fig. 2. *Ex vivo* IFN-γ responses to SARS-CoV-2 BA.1 and BA.2 mutation pools. A, Overview of variant-defining mutations in the spike protein described for the different variants of concern (VOC). **B-E,** Variant mutation-specific T-cell responses after complete vaccination (CV, two doses of either BNT162b2, mRNA-1273 and ChAdOx1 or one dose of Ad26.COV2.S, for heterologous vaccine regimens one dose of the vector vaccine ChAdOx1 followed by one dose of an mRNA vaccine), were assessed by interferon-gamma (IFN-γ) enzyme-linked immunospot (ELISpot) assays. **B,** Percentage of individuals with BA.1 mutation pool-specific *ex vivo* IFN-γ T-cell responses), and **C,** intensities of IFN-γ T-cell responses in terms of calculated spot counts () after mRNA, vector or heterologous (heterol.) vaccination, compared to COVID-19 convalescents (Conv.) and prepandemic (Pre.) donors. **D,** Percentage of individuals with BA.2 mutation pool-specific *ex vivo* IFN-γ T-cell responses, and **E,** intensities of IFN-γ T-cell responses in terms of calculated spot counts. Responders are represented by colored symbols, non-responders by clear symbols. Symbol shapes indicate the different vaccine products received by the donors. **C,E,** box plots represent the median with 25th and 75th percentiles with minimum and maximum whiskers. **B,D,** Fisher's exact test was used. **C,E,** Kruskal-Wallis test was used, if no p values are shown results were not significant. No., number.

profile in both vaccination cohorts after complete and booster vaccination with in particular positivity for IFN-γ (Fig. 4I,J; Fig. S8). Within the vaccine-induced TNF-producing spike-specific T cells, the proportion of $CD45RO^{+}CD4^{+}$ memory T cells showed a slight increase after booster vaccination compared to complete vaccination in both vaccination cohorts (mRNA: 78% vs. 82%, heterologous: 75% vs. 88%) (Fig. 4K, Fig. S9C-F). For vaccine-induced INF-γ-producing spike-specific $CD8^{+}$ T cells, this increase in memory T-cell response is only detected after heterologous vaccination (mRNA: 41% vs. 16%, heterologous: 0% vs. 14%, Fig. 4L).

In summary, the booster vaccination led to a significant increase of anti-spike IgG responses which show a marked decline 6 month after complete vaccination. In contrast anti-spike T-cell responses remained stable over time following complete vaccination with no

Fig. 3. *Ex vivo* characterization of spike-specific T-cell responses after complete vaccination. Spike-specific T-cell responses after complete vaccination (CV, two doses of either BNT162b2, mRNA-1273 or ChAdOx1, one dose of Ad26.COV2.S, or one dose of the vector vaccine ChAdOx1 followed by one dose of an mRNA vaccine for heterologous vaccine regimens) were characterized *ex vivo* by intracellular cytokine (interferon-gamma (IFN-γ), tumor necrosis factor (TNF)) and surface marker (CD107a) staining. **A,** Percentage of individuals with *ex vivo* $CD4^+$ (left panel), $CD8^+$ (middle panel) as well as both $CD4^+$ and $CD8^+$ (right panel) T-cell responses to the SARS-CoV-2 spike-specific peptide pools. **B,** Frequencies of spike-specific $CD4^+$ T cells after CV assessed *ex vivo*. **C,** Exemplary flow cytometry data of indicated cytokines and surface marker shown for $CD4^+$ T cells for one donor after CV (BNT162b2 x BNT162b2) with an mRNA vaccine. **D,** Proportion of samples with non-functional (0), mono-functional (1), bi-functional (2) or tri-functional (3) spike-specific $CD4^+$ T cells after CV. **E,** Frequencies of spike-specific $CD8^+$ T cells after CV assessed *ex vivo*. **F,** Exemplary flow cytometry data of indicated cytokines and surface marker shown for $CD8^+$ T cells for one donor after CV (BNT162b2 x BNT162b2) with an mRNA vaccine. **G,** Proportion of samples with non-functional (0), mono-functional (1), bi-functional (2) or tri-functional (3) spike-specific $CD8^+$ T cells after CV. T-cell responses were considered positive if the detected frequency of cytokine-positive $CD4^+$ or $CD8^+$ T cells was ≥3-fold higher than the frequency in the negative control and at least 0.1% of total $CD4^+$ or $CD8^+$ T cells. Responders are represented by colored symbols, non-responders by clear symbols. Symbol shapes indicate the different vaccine products received by the donors. **A,** Fisher's exact test. **B,E,** box plots show the median with 25th and 75th percentiles, whiskers represent minimum and maximum; Kruskal-Wallis test was used, if p values are not shown the results were not significant. FSC, forward scatter; Neg., negative control.

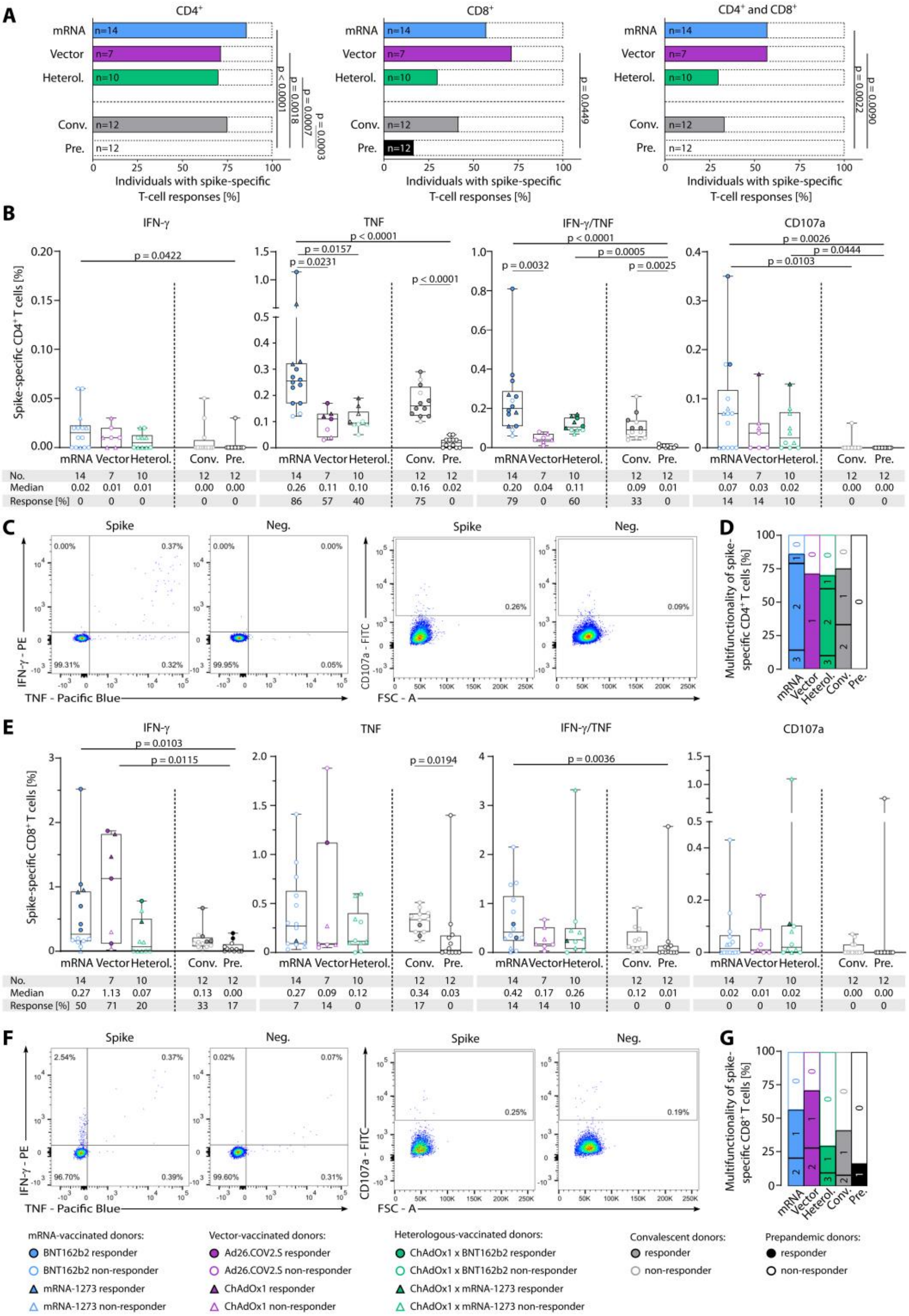

significant effect of booster vaccination on total intensity and frequency of T-cell responses and on cross-recognition of Omicron BA.1 and BA.2 mutations within the spike protein.

DISCUSSION

T-cell immunity is central for the control of viral infections. Whereas the role of anti-viral T-cell response is extensively studied during acute SARS-CoV-2 infection and COVID-19 (*13*, *15*, *19*, *20*), the induction of T cells upon vaccination with the different approved COVID-19 vaccines is studied less extensively (*12*, *17*). This study reports on T-cell immunity following complete and

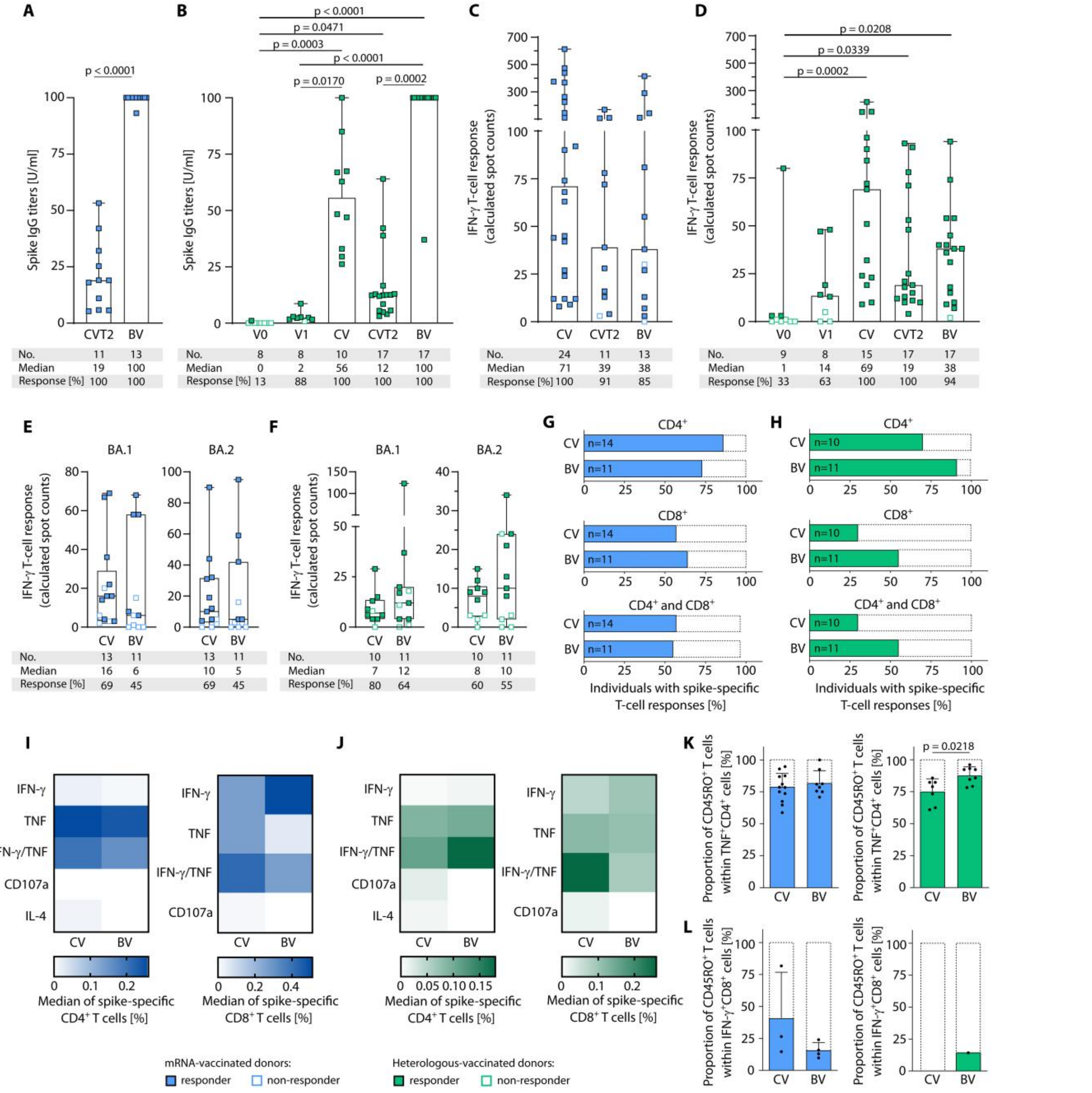

Fig. 4. T-cell and antibody responses of mRNA- and heterologous-vaccinated individuals after booster vaccination. A-D, Time course of spike antibody titers (**A,B**), and intensities of *ex vivo* interferon-gamma (IFN-γ) T-cell responses in terms of calculated spot counts were assessed by interferon-gamma (IFN-γ) enzyme-linked immunospot (ELISpot) assays targeting spike-specific peptide pools (**C,D**) after mRNA (**A,C**) and heterologous (heterol.) (**B,D**) vaccination, either before (V0), one month after first (V1) and complete vaccination (CV), six months after CV (CVT2) and one month after boost vaccination (BV). For results of paired samples from the same donors at each time point please refer to Fig. S9 (paired samples n = 8 for heterologous vaccination, n = 2 for mRNA vaccination). **E,F,** Intensities of *ex vivo* IFN-γ T-cell responses against the SARS-CoV-2 BA.1 and BA.2 mutation pools at CV and BV for mRNA and heterologous vaccinated donors, respectively. Responders are represented by colored symbols, non-responders by clear symbols. **G-L,** T-cell responses were characterized by *ex vivo* intracellular cytokine and surface marker staining. T-cell responses were considered positive if the detected frequency of cytokine-positive $CD4^+$ or $CD8^+$ T cells was ≥3-fold higher than the frequency in the negative control and minimum 0.1% of total $CD4^+$ or $CD8^+$ T cells. **G,H,** Percentage of individuals with $CD4^+$ (upper panel), $CD8^+$ (middle panel), as well as both $CD4^+$ and $CD8^+$ (lower panel) *ex vivo* T-cell responses to spike-specific peptide pools during the course of mRNA (**G**) and heterologous (**H**) vaccination. **I,J,** Heatmaps showing the percentages of cytokine and surface marker expressing $CD4^+$ and $CD8^+$ T cells *ex vivo* after CV and BV following mRNA (**I**) and heterologous (**J**) vaccination. **K,** Proportion of TNF^+CD4^+ spike-specific T cells expressing the T-cell memory marker CD45RO after CV (n = 12, n = 7) and BV (n = 8, n = 8) for mRNA- and heterologous-vaccinated donors, respectively. **L,** Proportion of $INF\text{-}\gamma^+CD8^+$ spike-specific T cells expressing the T-cell memory marker CD45RO after CV (n = 3, n = 0) and BV (n = 4, n = 1) for mRNA- and heterologous-vaccinated donors, respectively. **A,B,** Antibody titers are shown in U/ml (1 U/ml corresponds to 21.80 BAU/ml). **A-D,** Data are presented as scatter dot plots with median, whiskers show maximum. **E,F,** box plots show the median with 25th and 75th percentiles, whiskers represent minimum and maximum. **K,L,** Data are presented as scatter dot plots with mean, error bars indicates standard deviation. **A,K,L** Mann-Whitney test was used, **B-F,** Kruskal-Wallis test was used. **G,H**, Fisher's exact test was used, if no p values are shown results were not significant. No., number.

booster vaccination regimens in comparison to SARS-CoV-2 T-cell responses in convalescents and prepandemic donors.

In line with previous reports (*21*, *22*), the frequency and intensity of spike-specific T-cell responses were lower in vector-vaccinated donors compared to mRNA- and heterologous-vaccinated individuals, who showed comparable T-cell responses. Of note, the observed difference between vaccination regimens vanished after *in vitro* T-cell expansion, indicating potent expandability of vaccine-induced T cells upon virus encounter. Besides the expandability of virus-specific T cells (*23*), the diversity of T-cell responses, *i.e.* recognition of multiple T-cell epitopes, is shown to be central to combat viral disease including SARS-CoV-2 (*15*, *24*). We showed that vaccine-induced T cells responded to different peptide pools covering the whole spike protein indicating highly diverse T-cell immunity by the different vaccination regimens. Our data on expandability and broadness of vaccine-induced T-cell responses indicated that mRNA, vector and heterologous vaccination regimens can be recommended in the future to induce protective T-cell immunity.

Comparison with spike-specific T-cell responses induced in non-hospitalized convalescent individuals revealed similar frequency and intensity of T cells induced by different vaccination regimens. Of note, the phenotype and functionality of vaccine-induced $CD4^+$ and $CD8^+$ T cells also resembled those after natural infection (*25*). The induction of both $CD4^+$ and $CD8^+$ T cells has been shown to be central for effective T-cell immunity in infectious and malignant disease (*26*).

Cross-reactivity of T cells for different virus species or even amongst different pathogens is a well-known phenomenon postulated to enable heterologous immunity to a pathogen after exposure to a non-identical pathogen (*27*). In SARS-CoV-2, cross-reactive T cells are associated with protection against infection in COVID-19 contacts (*28*) and with enhanced immune responses upon infection and vaccination (*18*). Here, we showed high frequencies of spike-specific T-cell responses in a cohort of prepandemic, unexposed donors after *in vitro* T-cell expansion. In line with previous reports (*18*, *20*), the intensity and diversity of these preexisting T-cell responses were significantly lower than in convalescent and vaccinated individuals. Interestingly and in contrast to previous reports (*18*), we could also show cross-reactive T-cell responses against the Prot_S1 peptide pool, covering the complete N-terminal part of the S1 domain of the spike protein, which is described as less HCoV homologous than the C-terminal section, covered by the Prot_S peptide pool, indicating that cross-reactivity is not only based on sequence similarity but also on physiochemical and HLA-binding properties (*29*, *30*).

Application of a booster vaccination after complete vaccination shows beneficial effects in terms of protection from SARS-CoV-2 infection and severe courses of COVID-19 (*31*, *32*). In line with previous reports, we showed significant increase of IgG titers after booster vaccination for both, mRNA and heterologous vaccination (*33*). In contrast, frequency and intensity of T-cell responses was not significantly boosted by the additional vaccination; however, T-cell responses also did not exhibit such a marked decline after the complete vaccination compared to antibody responses. This is in line with reports after SARS-CoV-2 infection showing a rapid antibody decline and persistence of T-cell immunity (*9*). No differences between mRNA and heterologous vaccination were observed in terms of T-cell frequency, intensity and ability of $CD4^+$ T cell to produce multiple cytokines after booster vaccination. Of note, cytokine production in $CD8^+$ T cells was only boosted in donors that received three doses of mRNA vaccine. These data indicated that BV is of particular relevance for the amelioration of antiviral antibody activity, whereas robust T-cell immunity is already established after complete vaccination.

We further observed cross-recognition of the Omicron BA.1 and BA.2 mutated regions of the spike protein by vaccine-induced T cells after complete and booster vaccination for the majority of donors in the mRNA- and heterologous-vaccinated cohorts. This is in line with the cross-reactivity potential of SARS-CoV-2-specific T cells to HCoV (*18*, *28*) and provides the basis for reported conservation of vaccine-induced T-cell responses against different SARS-CoV-2 variants (*12*, *17*). This cross-reactivity is suggested to balance the lack of neutralizing antibodies targeting newly arising VOCs (*34*) and thus to prevent severe COVID-19 in vaccinees. These data on the cross-recognition potential of vaccine-induced T cells indicate that robust T-cell immunity toward Omicron variants is also induced from complete vaccination.

There are several limitations to our study. We had a limited number of samples available. Also, the vector vaccinated group is not the most relevant, as vector-based vaccines stopped being recommended by German governments in mid-2021 (*35*). The other main limitation is the restricted number of paired samples for the analysis over time.

Together, our work shows that complete vaccination against COVID-19 induces broad spike-specific $CD4^+$ and $CD8^+$ T-cell immunity by different vaccination regimens that resemble T-cell responses after natural SARS-CoV-2 infection. Moreover, booster vaccination seems of particular relevance for the amelioration of antiviral antibody activity, as T-cell responses are not markedly boosted by a third vaccination.

MATERIALS AND METHODS

Study design

This prospective cohort study was initiated in 2021 and describes T-cell responses in donors vaccinated with different COVID-19 vaccines after complete and booster vaccination (regimens described in more detail below), compared to convalescent and prepandemic donors. Starting January 2021, the German population was recommended to get vaccinated with the approved COVID-19 vaccines (BNT162b2, mRNA-1273, ChAdOx1 or Ad26.COV2.S), and volunteers were asked to participate in our study aiming to identify differences in T-cell responses following the different vaccination regimens. T-cell responses against the whole spike protein and against the Omicron BA.1 and BA.2 variant mutations were assessed. The control groups included samples collected from volunteer convalescents in 2020 after positive PCR test, and prepandemic samples collected before March 2017. No randomization was performed and blinding was not appropriate for this study. The methods and assays used were standardized to prevent batch effects. Data for the time point before and after first and complete vaccination of the same donor were obtained in the same assay and data before and after booster vaccination were obtained in the same assay.

Donors and blood samples

Peripheral blood mononuclear cells (PBMCs) from vaccinated donors, COVID-19 convalescents, and from prepandemic healthy volunteers, collected between August 2015 and March 2017 at the University Hospital Tübingen and the Cancer Research Department Rhein-Main (Hospital Nordwest), were isolated by density gradient centrifugation and stored at −80°C for short term storage or in liquid nitrogen until further use for subsequent T cell-based assays. Informed consent was obtained in accordance with the Declaration of Helsinki protocol. The study was performed according to the guidelines of the local ethics committees (179/2020/BO2, MC 288/2015, 2021–2305-evBO).

Donors vaccinated with different COVID-19 vaccination regimens

To assess spike-specific immune responses after vaccination blood samples from donors vaccinated with three different COVID-19 vaccine regimens were collected. The messenger ribonucleic acid (mRNA)-based vaccine cohort includes healthy volunteers vaccinated two (complete vaccination) to three times (booster vaccination) either with mRNA-1273 or with BNT162b2. The heterologous vaccination group received one dose of ChAdOx1 followed by one (complete vaccination) or two doses (booster vaccination) of either mRNA-1273 or BNT162b2. Donors of the vector-based vaccine group either received two doses of AZD1222 or one dose of JNJ-78436735 for complete vaccination. Donor characteristics and side effects after vaccination of the cohorts (n = 61) are provided in Table 1 and were assessed by questionnaire. Donors reporting headache, fever or shivering after vaccination where classified as donors with side effects.

SARS-CoV-2 convalescent individuals

To delineate differences of SARS-CoV-2 immune responses in vaccinated participants to immune responses after natural infection, a reference group of COVID-19 convalescent individuals, described previously (*20*) was used for comparison. SARS-CoV-2 infection was confirmed by real-time polymerase chain reaction (PCR) after nasopharyngeal swab. Sample collection for human COVID-19 convalescents (n = 16) was performed in July 2020, 94–130 days (median 117 days) after positive PCR. By the time of sample collection, the wildtype SARS-CoV-2 was circulating, variants of concern emerged at a later time point. Donor characteristics and COVID-19 symptoms were assessed by questionnaire. Details are provided in Table 2. Written informed consent was obtained in accordance with the Declaration of Helsinki protocol (179/2020/BO2).

IFN-γ ELISpot assay

ELISpot assays were performed *ex vivo* or following 12-day *in vitro* expansion. For *in vitro* expansion, PBMCs were pulsed with overlapping 15-mer peptide pools covering the entire spike protein (Miltenyi, PepTivator SARS-CoV-2 Prot_S, PepTivator SARS-CoV-2 Prot_S+, PepTivator SARS-CoV-2 Prot_S1, Fig. 1A) or the Omicron BA.1 and BA.2 mutated regions (Miltenyi, PepTivator SARS-CoV-2 Prot_S B.1.1.529/BA.1 Mutation Pool and PepTivator SARS-CoV-2 Prot_S B.1.1.529/BA.2 Mutation Pool) (0.02 nmol/peptide per mL) and cultured for 12 days adding 20 U/mL interleukin-2 (IL-2, Novartis) on days 3, 5, and 7. Peptide-stimulated (*in vitro* expanded) or freshly thawed (*ex vivo*) PBMCs were analyzed by IFN-γ ELISpot assay, as described previously (*20*). In brief, 100,000–300,000 cells per well were incubated in 96-well ELISpot plates coated with anti-IFN-γ antibody (clone 1-D1K, 2 μg/mL, MabTech, Cat# 3420–3-250, RRID: AB_907283) with peptide pools (0.01 nmol/peptide per mL). PHA (Sigma-Aldrich) served as positive control. An irrelevant HLA-DR-restricted control peptide (ETVITVDTKAAGKGK, FLNA_HUMAN1669–1683) in double-distilled water served as negative controls. After 24 h of incubation, spots were revealed with anti-IFN-γ biotinylated detection antibody (clone 7-B6–1, 0.3 μg/mL, MabTech, Cat# 3420–6-250, RRID: AB_907273), ExtrAvidin-Alkaline Phosphatase (1:1,000 dilution, Sigma-Aldrich), and BCIP/NBT (5-bromo-4-chloro-3-indolyl-phosphate/nitro-blue tetrazolium chloride, Sigma-Aldrich). Spots were counted using an ImmunoSpot S6 analyzer (CTL). T-cell responses were considered positive, and donors as responders, if the mean spot count of the technical replicates normalized to 300,000 cells was at least 3 spots *ex vivo* and 6 spots after 12-day *in vitro* expansion and 3-fold higher than the mean spot count of the negative control normalized to 300,000 cells (*15*, *36*). Intensity of T-cell responses is depicted as calculated spot counts, which represent the sum of mean spot count normalized to 300,000 cells for all three tested spike-specific peptide pools subtracting the normalized mean spot count of the respective negative control.

Intracellular cytokine and cell surface marker staining

Peptide-specific T cells were characterized by cell surface marker and intracellular cytokine staining (ICS) as previously described (*20*). In brief, 250,000 to 1,000,000 PBMCs were incubated over 12 to 14 hours with the 15-mer peptide pools covering the entire spike protein or the negative control peptide, Brefeldin A (Sigma-Aldrich), and GolgiStop (BD Biosciences). PMA and ionomycin (Sigma-Aldrich, Cat#L1668) served as positive control, for the *ex vivo* ICS SEB (Sigma-Aldrich, Cat#S4881) was used as additional positive control. Staining was performed using Cytofix/Cytoperm solution (BD), Zombie Aqua (for *ex vivo* samples, 1:200 dilution, BioLegend), APC/Cy7 anti-human CD4 (1:100 dilution, BioLegend, Cat#300518, RRID: AB_314086), PE/Cy7 anti-human CD8 (1:400 dilution, Beckman Coulter, Cat#737661, RRID: AB_1575980), Pacific Blue anti-human tumor necrosis factor (TNF, 1:120 dilution, BioLegend, Cat#502920, RRID: AB_528965), FITC anti-human CD107a (1:100 dilution, BioLegend, Cat#328606, RRID: AB_1186036), PE anti-human IFN-γ monoclonal antibodies (1:200 dilution, BioLegend, Cat#506507, RRID: AB_315440), APC anti-human CD45RO (1:100 dilution, BioLegend, Cat#304210, RRID: AB_314426), PE-Dazzle 594 anti-human IL-4 (1:25 dilution, BioLegend, Cat#500832, RRID: AB_2564036). *Ex vivo* samples were analyzed on a FACS LSRFortessa (BD, gating strategy Fig. S10). In this study, a T helper 1 (T_H1) response was defined as cells producing IFN-γ and tumor necrosis factor, and a T helper 2 response (T_H2) as cells producing IL-4. T-cell responses were considered positive if the detected frequency of cytokine-positive $CD4^+$ or $CD8^+$ T cells was ≥3-fold higher than the frequency in the negative control and minimum 0.1%. Frequency of cytokine-positive cells was corrected for background by subtraction of the respective negative control values. Negative values were set to zero.

SARS-CoV-2 anti-spike and anti-nucleocapsid antibody testing

The Siemens SARS-CoV-2 IgG (SCOVG) assay was performed on an automated ADVIA Centaur XPT system (Siemens Healthineers) according to the manufacturer's instructions. The immunoassay detects anti-SARS-CoV-2 IgG antibodies (SCOVG) directed against the S1 domain of the viral spike protein (including the immunologically relevant receptor binding domain). The Elecsys Assay from Roche detecting high affinity antibodies (including IgG) directed against the nucleocapsid protein of SARS-CoV-2 was performed according to the manufacturer's instructions for samples collected at the University Hospital Tübingen. Results are reported in index values for the Roche assay and the SCOVG assay. For the latter, an index value of 1 corresponds to one unit per milliliter (U/ml), 1 U/ml can be converted to 21.80 binding antibody units (BAU)/ml according to the manufacturer. The final interpretation of positivity is determined by an antibody titer ≥1.0 U/ml given by the manufacturer. Values <0.1 were set to 0.1. 100 was the highest measurable index value with the SCOVG assay. Quality control was performed following the manufacturer's instructions on each day of testing.

Software and statistical analysis

Flow cytometric data was analyzed using FlowJo 10.7.1 (BD). Graphs were plotted using Inkscape 1.1 and GraphPad Prism 9.2.0. Statistical analyses were conducted using GraphPad Prism 9.2.0. Data are displayed as mean ± SD, box plots as median with 25% or 75% quantiles and min/max whiskers. Continuous data were tested for distribution and individual groups were tested by use of two-sided Fisher's exact test, unpaired t-test, unpaired Mann-Whitney-U-test, Kruskal-Wallis test, or paired Wilcoxon signed rank test and Friedman test, all performed as two-sided tests. Correlation was tested using Spearman test and linear regression. P values of <0.05 were considered statistically significant.

Supplementary Materials

This PDF file includes:

Figs. to S10
Table S1

Other Supplementary Material for this manuscript includes the following:

Table S2

View/request a protocol for this paper from *Bio-protocol*.

REFERENCES AND NOTES

1. M. N. Ramasamy, A. M. Minassian, K. J. Ewer, A. L. Flaxman, P. M. Folegatti, D. R. Owens, M. Voysey, P. K. Aley, B. Angus, G. Babbage, S. Belij-Rammerstorfer, L. Berry, S. Bibi, M. Bittaye, K. Cathie, H. Chappell, S. Charlton, P. Cicconi, E. A. Clutterbuck, R. Colin-Jones, C. Dold, K. R. W. Emary, S. Fedosyuk, M. Fuskova, D. Gbesemete, C. Green, B. Hallis, M. M. Hou, D. Jenkin, C. C. D. Joe, E. J. Kelly, S. Kerridge, A. M. Lawrie, A. Lelliott, M. N. Lwin, R. Makinson, N. G. Marchevsky, Y. Mujadidi, A. P. S. Munro, M. Pacurar, E. Plested, J. Rand, T. Rawlinson, S. Rhead, H. Robinson, A. J. Ritchie, A. L. Ross-Russell, S. Saich, N. Singh, C. C. Smith, M. D. Snape, R. Song, R. Tarrant, Y. Themistocleous, K. M. Thomas, T. L. Villafana, S. C. Warren, M. E. E. Watson, A. D. Douglas, A. V. S. Hill, T. Lambe, S. C. Gilbert, S. N. Faust, A. J. Pollard; Oxford COVID Vaccine Trial Group, Safety and immunogenicity of ChAdOx1 nCoV-19 vaccine administered in a prime-boost regimen in young and old adults (COV002): A single-blind, randomised, controlled, phase 2/3 trial. *Lancet* **396**, 1979–1993 (2021).
2. F. P. Polack, S. J. Thomas, N. Kitchin, J. Absalon, A. Gurtman, S. Lockhart, J. L. Perez, G. Perez Marc, E. D. Moreira, C. Zerbini, R. Bailey, K. A. Swanson, S. Roychoudhury, K. Koury, P. Li, W. V. Kalina, D. Cooper, R. W. Frenck Jr., L. L. Hammitt, O. Tureci, H. Nell, A. Schaefer, S. Unal, D. B. Tresnan, S. Mather, P. R. Dormitzer, U. Sahin, K. U. Jansen, W. C. Gruber; C4591001 Clinical Trial Group, Safety and efficacy of the BNT162b2 mRNA Covid-19 vaccine. *N. Engl. J. Med.* **383**, 2603–2615 (2020).
3. L. R. Baden, H. M. El Sahly, B. Essink, K. Kotloff, S. Frey, R. Novak, D. Diemert, S. A. Spector, N. Rouphael, C. B. Creech, J. McGettigan, S. Khetan, N. Segall, J. Solis, A. Brosz, C. Fierro, H. Schwartz, K. Neuzil, L. Corey, P. Gilbert, H. Janes, D. Follmann, M. Marovich, J. Mascola, L. Polakowski, J. Ledgerwood, B. S. Graham, H. Bennett, R. Pajon, C. Knightly, B. Leav, W. Deng, H. Zhou, S. Han, M. Ivarsson, J. Miller, T. Zaks; COVE Study Group, Efficacy and safety of the mRNA-1273 SARS-CoV-2 vaccine. *N. Engl. J. Med.* **384**, 403–416 (2021).
4. J. Sadoff, G. Gray, A. Vandebosch, V. Cardenas, G. Shukarev, B. Grinsztejn, P. A. Goepfert, C. Truyers, H. Fennema, B. Spiessens, K. Offergeld, G. Scheper, K. L. Taylor, M. L. Robb, J. Treanor, D. H. Barouch, J. Stoddard, M. F. Ryser, M. A. Marovich, K. M. Neuzil, L. Corey, N. Cauwenberghs, T. Tanner, K. Hardt, J. Ruiz-Guinazu, M. Le Gars, H. Schuitemaker, J. Van Hoof, F. Struyf, M. Douoguih; ENSEMBLE Study Group, Safety and efficacy of single-dose Ad26.COV2.S vaccine against Covid-19. *N. Engl. J. Med.* **384**, 2187–2201 (2021).
5. Our World in Data, COVID-19 vaccine doses administered by manufacturer, European Union (2022); https://ourworldindata.org/covid-vaccinations#which-vaccines-have-been-administered-in-each-country.
6. Center for Disease Control and Prevention, U.S. COVID-19 Vaccine Administration by Vaccine Type (2022); https://covid.cdc.gov/covid-data-tracker/#vaccinations_vacc-people-additional-dose-totalpop.
7. Government of Canada, Cumulative number of COVID-19 vaccine doses administered in Canada by vaccine product and dose number, as of October 9, 2022 (2022); https://health-infobase.canada.ca/covid-19/vaccine-administration/.
8. A. Greinacher, T. Thiele, T. E. Warkentin, K. Weisser, P. A. Kyrle, S. Eichinger, Thrombotic thrombocytopenia after ChAdOx1 nCov-19 vaccination. *N. Engl. J. Med.* **384**, 2092–2101 (2021).
9. E. G. Levin, Y. Lustig, C. Cohen, R. Fluss, V. Indenbaum, S. Amit, R. Doolman, K. Asraf, E. Mendelson, A. Ziv, C. Rubin, L. Freedman, Y. Kreiss, G. Regev-Yochay, Waning immune humoral response to BNT162b2 Covid-19 vaccine over 6 months. *N. Engl. J. Med.* **385**, e84 (2021).
10. N. Barda, N. Dagan, C. Cohen, M. A. Hernan, M. Lipsitch, I. S. Kohane, B. Y. Reis, R. D. Balicer, Effectiveness of a third dose of the BNT162b2 mRNA COVID-19 vaccine for preventing severe outcomes in Israel: An observational study. *Lancet* **398**, 2093–2100 (2021).
11. D. S. Khoury, D. Cromer, A. Reynaldi, T. E. Schlub, A. K. Wheatley, J. A. Juno, K. Subbarao, S. J. Kent, J. A. Triccas, M. P. Davenport, Neutralizing antibody levels are highly predictive of immune protection from symptomatic SARS-CoV-2 infection. *Nat. Med.* **27**, 1205–1211 (2021).
12. A. Tarke, C. H. Coelho, Z. Zhang, J. M. Dan, E. D. Yu, N. Methot, N. I. Bloom, B. Goodwin, E. Phillips, S. Mallal, J. Sidney, G. Filaci, D. Weiskopf, R. da Silva Antunes, S. Crotty, A. Grifoni, A. Sette, SARS-CoV-2 vaccination induces immunological T cell memory able to cross-recognize variants from Alpha to Omicron. *Cell* **185**, 847–859.e11 (2022).
13. A. T. Tan, M. Linster, C. W. Tan, N. Le Bert, W. N. Chia, K. Kunasegaran, Y. Zhuang, C. Y. L. Tham, A. Chia, G. J. D. Smith, B. Young, S. Kalimuddin, J. G. H. Low, D. Lye, L. F. Wang, A. Bertoletti, Early induction of functional SARS-CoV-2-specific T cells associates with rapid viral clearance and mild disease in COVID-19 patients. *Cell Rep.* **34**, 108728 (2021).
14. D. Geers, M. C. Shamier, S. Bogers, G. den Hartog, L. Gommers, N. N. Nieuwkoop, K. S. Schmitz, L. C. Rijsbergen, J. A. T. van Osch, E. Dijkhuizen, G. Smits, A. Comvalius, D. van Mourik, T. G. Caniels, M. J. van Gils, R. W. Sanders, B. B. Oude Munnink, R. Molenkamp, H. J. de Jager, B. L. Haagmans, R. L. de Swart, M. P. G. Koopmans, R. S. van Binnendijk, R. D. de Vries, C. H. GeurtsvanKessel, SARS-CoV-2 variants of concern partially escape humoral but not T-cell responses in COVID-19 convalescent donors and vaccinees. *Sci. Immunol.* **6**, eabj1750 (2021).
15. T. Bilich, A. Nelde, J. S. Heitmann, Y. Maringer, M. Roerden, J. Bauer, J. Rieth, M. Wacker, A. Peter, S. Horber, D. Rachfalski, M. Marklin, S. Stevanovic, H. G. Rammensee, H. R. Salih, J. S. Walz, T cell and antibody kinetics delineate SARS-CoV-2 peptides mediating long-term immune responses in COVID-19 convalescent individuals. *Sci. Transl. Med.* **13**, eabf7517 (2021).
16. R. Rose, F. Neumann, O. Grobe, T. Lorentz, H. Fickenscher, A. Krumbholz, Humoral immune response after different SARS-CoV-2 vaccination regimens. *BMC Med.* **20**, 31 (2022).
17. C. H. GeurtsvanKessel, D. Geers, K. S. Schmitz, A. Z. Mykytyn, M. M. Lamers, S. Bogers, S. Scherbeijn, L. Gommers, R. S. G. Sablerolles, N. N. Nieuwkoop, L. C. Rijsbergen, L. L. A. van Dijk, J. de Wilde, K. Alblas, T. I. Breugem, B. J. A. Rijnders, H. de Jager, D. Weiskopf, P. H. M. van der Kuy, A. Sette, M. P. G. Koopmans, A. Grifoni, B. L. Haagmans, R. D. de Vries, Divergent SARS-CoV-2 Omicron-reactive T and B cell responses in COVID-19 vaccine recipients. *Sci. Immunol.* **7**, eabo2202 (2022).

18. L. Loyal, J. Braun, L. Henze, B. Kruse, M. Dingeldey, U. Reimer, F. Kern, T. Schwarz, M. Mangold, C. Unger, F. Dorfler, S. Kadler, J. Rosowski, K. Gurcan, Z. Uyar-Aydin, M. Frentsch, F. Kurth, K. Schnatbaum, M. Eckey, S. Hippenstiel, A. Hocke, M. A. Muller, B. Sawitzki, S. Miltenyi, F. Paul, M. A. Mall, H. Wenschuh, S. Voigt, C. Drosten, R. Lauster, N. Lachman, L. E. Sander, V. M. Corman, J. Rohmel, L. Meyer-Arndt, A. Thiel, C. Giesecke-Thiel, Cross-reactive CD4$^+$ T cells enhance SARS-CoV-2 immune responses upon infection and vaccination. *Science* **374**, eabh1823 (2021).
19. J. Braun, L. Loyal, M. Frentsch, D. Wendisch, P. Georg, F. Kurth, S. Hippenstiel, M. Dingeldey, B. Kruse, F. Fauchere, E. Baysal, M. Mangold, L. Henze, R. Lauster, M. A. Mall, K. Beyer, J. Röhmel, S. Voigt, J. Schmitz, S. Miltenyi, I. Demuth, M. A. Müller, A. Hocke, M. Witzenrath, N. Suttorp, F. Kern, U. Reimer, H. Wenschuh, C. Drosten, V. M. Corman, C. Giesecke-Thiel, L. E. Sander, A. Thie, SARS-CoV-2-reactive T cells in healthy donors and patients with COVID-19. *Nature* **587**, 270–274 (2020).
20. A. Nelde, T. Bilich, J. S. Heitmann, Y. Maringer, H. R. Salih, M. Roerden, M. Lubke, J. Bauer, J. Rieth, M. Wacker, A. Peter, S. Horber, B. Traenkle, P. D. Kaiser, U. Rothbauer, M. Becker, D. Junker, G. Krause, M. Strengert, N. Schneiderhan-Marra, M. F. Templin, T. O. Joos, D. J. Kowalewski, V. Stos-Zweifel, M. Fehr, A. Rabsteyn, V. Mirakaj, J. Karbach, E. Jager, M. Graf, L. C. Gruber, D. Rachfalski, B. Preuss, I. Hagelstein, M. Marklin, T. Bakchoul, C. Gouttefangeas, O. Kohlbacher, R. Klein, S. Stevanovic, H. G. Rammensee, J. S. Walz, SARS-CoV-2-derived peptides define heterologous and COVID-19-induced T cell recognition. *Nat. Immunol.* **22**, 74–85 (2021).
21. T. Schmidt, V. Klemis, D. Schub, J. Mihm, F. Hielscher, S. Marx, A. Abu-Omar, L. Ziegler, C. Guckelmus, R. Urschel, S. Schneitler, S. L. Becker, B. C. Gartner, U. Sester, M. Sester, Immunogenicity and reactogenicity of heterologous ChAdOx1 nCoV-19/mRNA vaccination. *Nat. Med.* **27**, 1530–1535 (2021).
22. D. Hillus, T. Schwarz, P. Tober-Lau, K. Vanshylla, H. Hastor, C. Thibeault, S. Jentzsch, E. T. Helbig, L. J. Lippert, P. Tscheak, M. L. Schmidt, J. Riege, A. Solarek, C. von Kalle, C. Dang-Heine, H. Gruell, P. Kopankiewicz, N. Suttorp, C. Drosten, H. Bias, J. Seybold; EICOV/COVIM Study Group, F. Klein, F. Kurth, V. M. Corman, L. E. Sander, Safety, reactogenicity, and immunogenicity of homologous and heterologous prime-boost immunisation with ChAdOx1 nCoV-19 and BNT162b2: A prospective cohort study. *Lancet Respir. Med.* **9**, 1255–1265 (2021).
23. T. M. Wilkinson, C. K. Li, C. S. Chui, A. K. Huang, M. Perkins, J. C. Liebner, R. Lambkin-Williams, A. Gilbert, J. Oxford, B. Nicholas, K. J. Staples, T. Dong, D. C. Douek, A. J. McMichael, X.-N. Xu, Preexisting influenza-specific CD4+ T cells correlate with disease protection against influenza challenge in humans. *Nat. Med.* **18**, 274–280 (2012).
24. A. C. Tan, N. L. La Gruta, W. Zeng, D. C. Jackson, Precursor frequency and competition dictate the HLA-A2-restricted CD8+ T cell responses to influenza A infection and vaccination in HLA-A2.1 transgenic mice. *J. Immunol.* **187**, 1895–1902 (2011).
25. L. B. Rodda, J. Netland, L. Shehata, K. B. Pruner, P. A. Morawski, C. D. Thouvenel, K. K. Takehara, J. Eggenberger, E. A. Hemann, H. R. Waterman, M. L. Fahning, Y. Chen, M. Hale, J. Rathe, C. Stokes, S. Wrenn, B. Fiala, L. Carter, J. A. Hamerman, N. P. King, M. Gale Jr., D. J. Campbell, D. J. Rawlings, M. Pepper, Functional SARS-CoV-2-specific immune memory persists after mild COVID-19. *Cell* **184**, 169–183.e17 (2021).
26. D. J. Shedlock, H. Shen, Requirement for CD4 T cell help in generating functional CD8 T cell memory. *Science* **300**, 337–339 (2003).
27. G. Petrova, A. Ferrante, J. Gorski, Cross-reactivity of T cells and its role in the immune system. *Crit. Rev. Immunol.* **32**, 349–372 (2012).
28. R. Kundu, J. S. Narean, L. Wang, J. Fenn, T. Pillay, N. D. Fernandez, E. Conibear, A. Koycheva, M. Davies, M. Tolosa-Wright, S. Hakki, R. Varro, E. McDermott, S. Hammett, J. Cutajar, R. S. Thwaites, E. Parker, C. Rosadas, M. McClure, R. Tedder, G. P. Taylor, J. Dunning, A. Lalvani, Cross-reactive memory T cells associate with protection against SARS-CoV-2 infection in COVID-19 contacts. *Nat. Commun.* **13**, 80 (2022).
29. O. Y. Borbulevych, K. H. Piepenbrink, B. E. Gloor, D. R. Scott, R. F. Sommese, D. K. Cole, A. K. Sewell, B. M. Baker, T cell receptor cross-reactivity directed by antigen-dependent tuning of peptide-MHC molecular flexibility. *Immunity* **31**, 885–896 (2009).
30. Y. Yin, R. A. Mariuzza, The multiple mechanisms of T cell receptor cross-reactivity. *Immunity* **31**, 849–851 (2009).
31. N. Andrews, J. Stowe, F. Kirsebom, S. Toffa, R. Sachdeva, C. Gower, M. Ramsay, J. Lopez Bernal, Effectiveness of COVID-19 booster vaccines against COVID-19-related symptoms, hospitalization and death in England. *Nat. Med.* **28**, 831–837 (2022).
32. Y. M. Bar-On, Y. Goldberg, M. Mandel, O. Bodenheimer, L. Freedman, N. Kalkstein, B. Mizrahi, S. Alroy-Preis, N. Ash, R. Milo, A. Huppert, Protection of BNT162b2 vaccine booster against Covid-19 in Israel. *N. Engl. J. Med.* **385**, 1393–1400 (2021).
33. J. Yu, A. Y. Collier, M. Rowe, F. Mardas, J. D. Ventura, H. Wan, J. Miller, O. Powers, B. Chung, M. Siamatu, N. P. Hachmann, N. Surve, F. Nampanya, A. Chandrashekar, D. H. Barouch, Neutralization of the SARS-CoV-2 omicron BA.1 and BA.2 variants. *N. Engl. J. Med.* **386**, 1579–1580 (2022).
34. World Health Organization, Tracking SARS-CoV-2 variants (2022); https://www.who.int/activities/tracking-SARS-CoV-2-variants.
35. Bundesregierung, AstraZeneca vaccine primarily for over-60s (2021); https://www.bundesregierung.de/breg-en/news/astrazeneca-vaccine-primarily-for-over-60s-1884412.
36. I. Cassaniti, F. Bergami, E. Percivalle, E. Gabanti, J. C. Sammartino, A. Ferrari, K. M. G. Adzasehoun, F. Zavaglio, P. Zelini, G. Comolli, A. Sarasini, A. Piralla, A. Ricciardi, V. Zuccaro, F. Maggi, F. Novazzi, L. Simonelli, L. Varani, D. Lilleri, F. Baldanti, Humoral and cell-mediated response against SARS-CoV-2 variants elicited by mRNA vaccine BNT162b2 in healthcare workers: a longitudinal observational study. *Clin. Microbiol. Infect.* **28**, 301.e1–301.e8 (2022).

Acknowledgments: We thank Ulrike Schmidt, Hannah Zug, Richard Schaad, Jonas Rieth, Iris Schäfer, Cécile Gouttefangeas and Jonas Heitmann for technical support and project coordination. This work is licensed under a Creative Commons Attribution 4.0 International (CC BY 4.0) license, which permits unrestricted use, distribution, and reproduction in any medium, provided the original work is properly cited. To view a copy of this license, visit http://creativecommons.org/licenses/by/4.0/. This license does not apply to figures/photos/artwork or other content included in the article that is credited to a third party; obtain authorization from the rights holder before using such material. **Funding:** This work was supported by the Ministry of Science, Research and the Arts Baden-Württemberg,Germany (MWK, Sonderfördermassnahme COVID-19, TÜ17) (J.S.W.), the Federal Agency of Science, Research and the Arts, Germany (BMBF, FKZ:01KI20130 and FKZ:16LW0004K) (J.S.W.), the Deutsche Forschungsgemeinschaft (DFG, German Research Foundation, Grant WA 4608/1-2) (J.S.W.), the Deutsche Forschungsgemeinschaft under Germany's Excellence Strategy (Grant EXC2180 390900677) (J.S.W.), the German Cancer Consortium (DKTK) (J.S.W.), the Wilhelm Sander Stiftung (Grant 2016.177.2) (J.S.W.) and the José Carreras Leukämie-Stiftung (Grant DJCLS 05 R/2017) (J.S.W.). **Author contributions:** Y.M., A.N. and J.S.W. were involved in the design of the overall study and strategy. Y.M. performed the T cell experiments. Spike-specific IgG were analyzed by S.H., A.P.. S.S., J.K., E.J, J.S.W. conducted donor data and sample collection. Y.M., A.N., J.S. and J.S.W. analyzed the data. Y.M., A.N. and J.S.W. drafted the manuscript. All authors supported the creation of the manuscript by review and editing. J.S.W. supervised the study. **Competing interests:** Authors declare that they have no competing interests. **Data and materials availability:** All data necessary to evaluate the conclusions in this study are present in the manuscript or the Supplementary Materials.

Submitted 22 June 2022
Resubmitted 02 September 2022
Accepted 25 October 2022
Published First Release 1 November 2022
10.1126/sciimmunol.add3899
