# Supplementary material for: Durable spike-specific T cell responses after different COVID-19 vaccination regimens are not further enhanced by booster vaccination
Source: Sci Immunol. 2022 Dec 16;7(78):eadd3899. doi: 10.1126/sciimmunol.add3899 (PMC9798886; doi:10.1126/sciimmunol.add3899)
Supplement: Supplementary file 2 — Figs. to S10 Table S1 [file sciimmunol.add3899_sm.pdf]

Science Immunology

AAAS

Supplementary Materials for

Durable spike-specific T cell responses after different COVID-19 vaccination regimens are not further enhanced by booster vaccination

Yacine Maringer *et al.*

Corresponding author: Juliane S. Walz, juliane.walz@med.uni-tuebingen.de

Sci. Immunol. 0, eadd3899 (2022)
DOI: 10.1126/sciimmunol.add3899

The PDF file includes:

Figs. S1 to S10
Table S1

Other Supplementary Materials for this manuscript includes the following:

Table S2

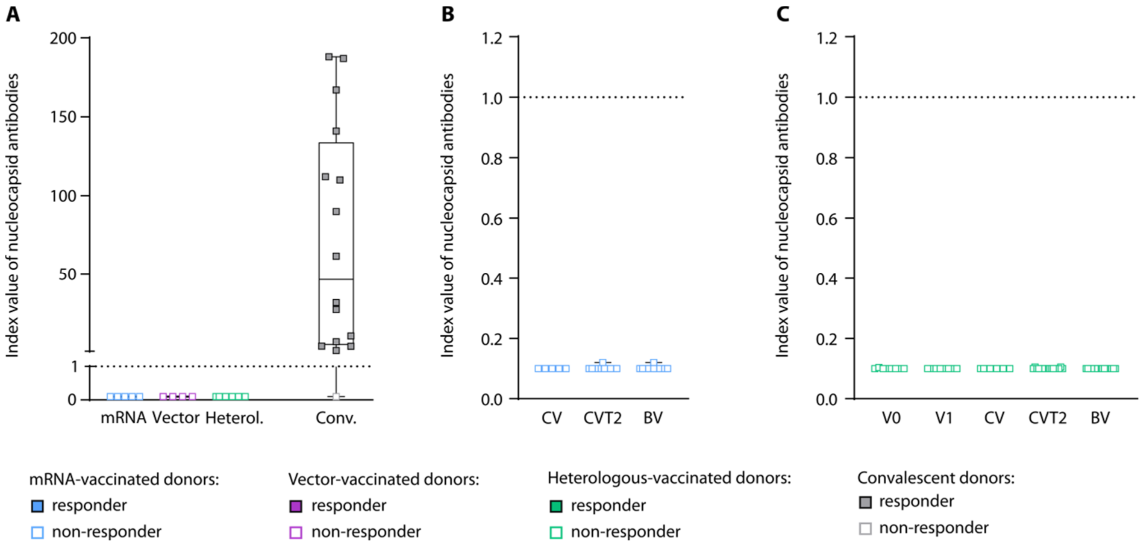

Fig. S1: Index values of nucleocapsid antibodies in vaccinated donors. Index values of nucleocapsid antibodies were assessed after complete vaccination (CV) for mRNA- (n = 5), vector- (n = 4) and heterologous- (n = 10) vaccinated donors (**A**), for the mRNA cohort after CV (n = 5), six months after CV (CVT2, n = 11) and after boost vaccination (BV, n = 12) (**B**), as well as for the heterologous cohort before vaccination (V0, n = 8), after first vaccination (V1, n = 8), after CV (n = 10), after CVT2 (n = 17) and after BV (n = 17) (**C**). The dashed line marks the nucleocapsid index value threshold for positivity. Responders are represented by colored symbols, non-responders by clear symbols. Results are represented as box plots showing median with 25th and 75th percentiles, whiskers represent minimum and maximum.

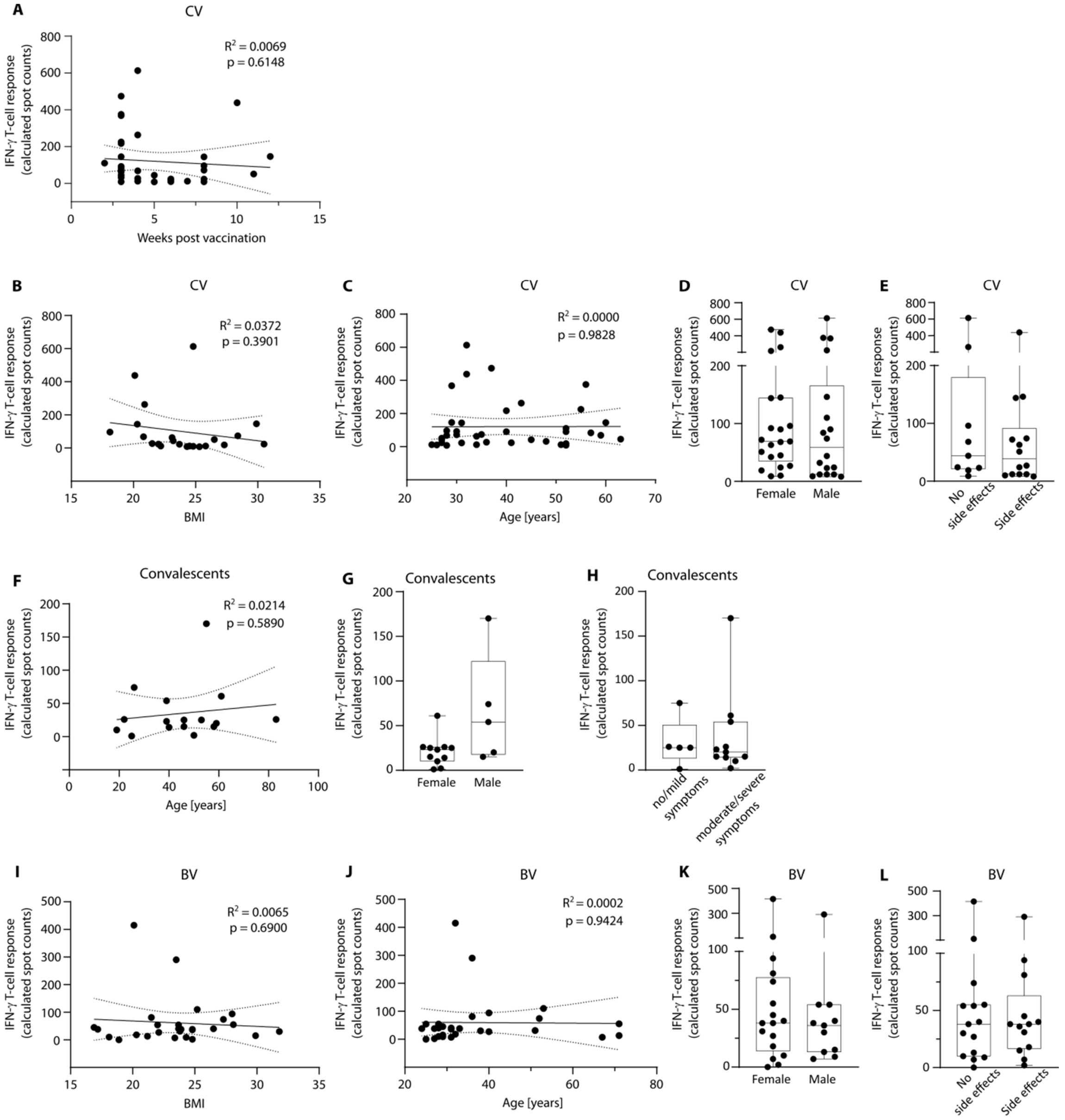

Fig. S2: Spike-specific T-cell responses after vaccination and in convalescents according to demographics and symptoms. A-L, Interferon-gamma (IFN-γ) T-cell responses were assessed using *ex vivo* ELISpot assays. Intensities of T-cell responses are depicted in terms of calculated spot counts. **A,** Correlation of the intensity of spike-specific IFN-γ T-cell responses after complete vaccination (CV, two doses of either BNT162b2 or mRNA-1273, or one dose of the vector vaccine ChAdOx1 followed by one dose of an mRNA vaccine) with time post vaccination (n = 39). **B,C**, Correlation of the intensity of spike-specific IFN-γ T-cell responses

after complete vaccination (CV) with BMI (**B**, n = 22) and age (**C**, n = 39). **D,E**, Comparison of the intensity of spike-specific IFN-γ T-cell responses after CV according to gender (**D**, n = 39) and side effects (headache, fever, shivering) after vaccination (**E**, n = 23). **F**, Correlation of the intensity of spike-specific IFN-γ T-cell responses with age for COVID-19 convalescents (n = 16). **G,H,** Comparison of the intensity of spike-specific IFN-γ T-cell response in convalescent donors according to gender (**G**, n = 16) and to clinical symptoms (no/mild vs. moderate/severe) during COVID-19 (**H**, n = 16). **I,J**, Correlation of the intensity of spike-specific IFN-γ T-cell responses after boost vaccination (BV) with BMI (**I**, n = 27) or age (**J**, n = 28). **K,L**, Comparison of the intensity of spike-specific IFN-γ T-cell responses after BV according to gender (**K**, n = 28) and side effects (headache, fever, shivering) after vaccination (**L**, n = 28). **A-C,F,I,J**, dotted lines show the 95% confidence level, R^2 and p value for linear regression are shown. **D,E,G,H,K,L,** box plots show median with 25th and 75th percentiles, whiskers represent minimum and maximum. **D,E,G,H,K,L,** Kruskal-Wallis test was used; if no p values are shown results were not significant.

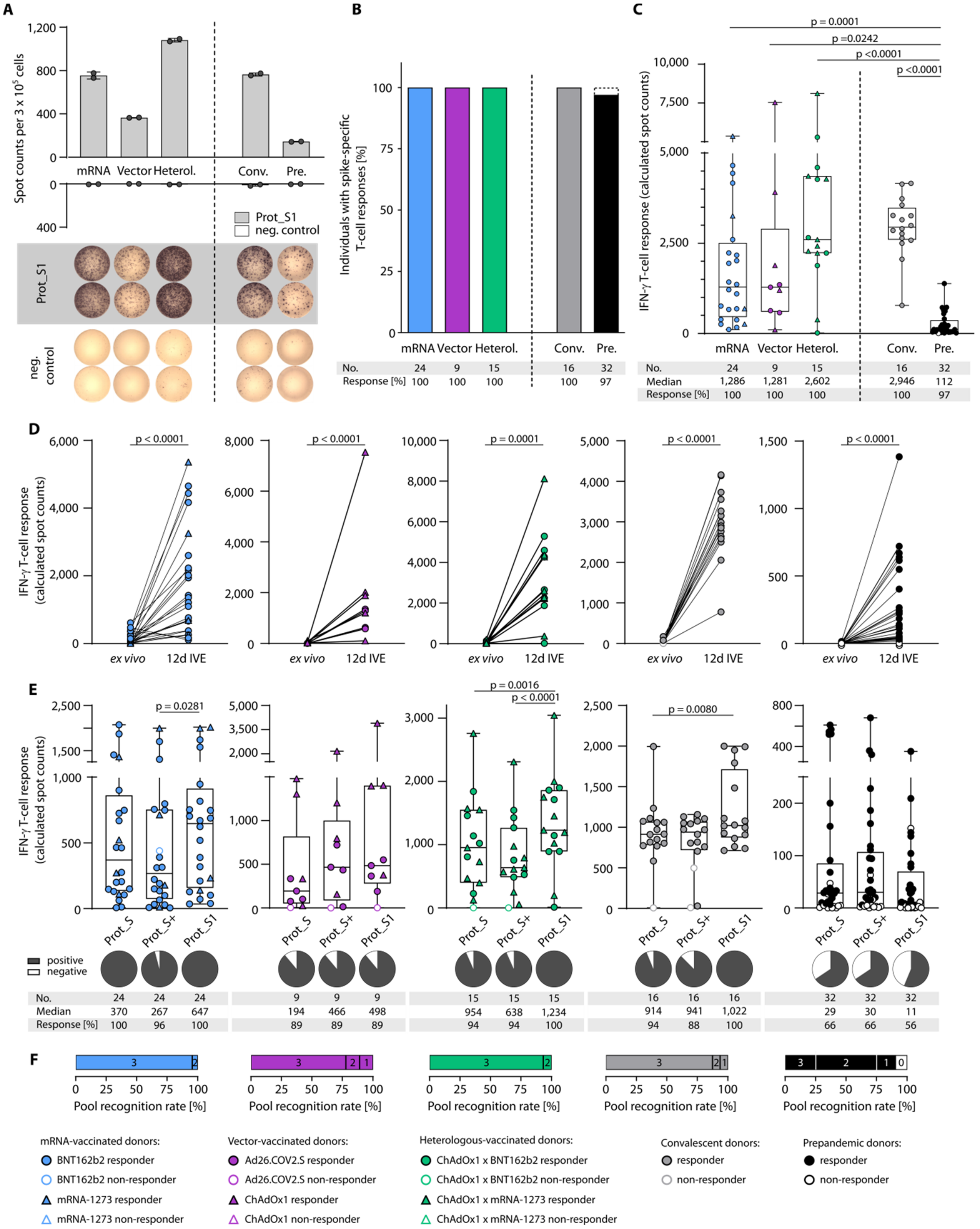

Fig. S3: Immune responses to the SARS-CoV-2 spike-specific peptide pools after complete vaccination following 12-day *in vitro* expansion. A, Representative example of interferon-gamma (IFN-γ) T-cell responses to the Prot_S1 peptide pool compared to a negative (neg.) control peptide, evaluated by IFN-γ ELISpot following 12-day *in vitro* T-cell expansion after complete vaccination (two doses of either BNT162b2, mRNA-1273 or

ChAdOx1, one dose of Ad26.COV2.S, or one dose of the vector vaccine ChAdOx1 followed by one dose of an mRNA vaccine for heterologous vaccine regimens), showing the duplicates for one donor of each cohort. **B,C,** Percentage of individuals with IFN-γ T-cell responses (**B**), and intensities of T-cell responses in terms of calculated spot counts (**C**) targeting the spike peptide pools after 12-day *in vitro* T-cell expansion following mRNA, vector or heterologous (heterol.) vaccination, in comparison to COVID-19 convalescents (Conv.) and prepandemic (Pre.) donors. **D,** Spike-specific IFN-γ T-cell responses assessed in the different cohorts *ex vivo* and after 12-day *in vitro* expansion (IVE). **E,** Intensities of IFN-γ T-cell response shown separately for the distinct spike-specific peptide pools. **F,** Proportion of individuals with responses to all three, two, one or none of the spike peptide pools. Responders are represented by colored symbols, non-responders by clear symbols. Symbol shapes indicate the different vaccine products received by the donors. In **B,C,** box plots represent median with 25th and 75th percentiles with minimum and maximum whiskers. **B,** Fisher's exact test was used, **C,** Kruskal-Wallis test was used, **D,** Wilcoxon test was applied, **E,** Friedman test was used, if no p values are shown results were not significant.

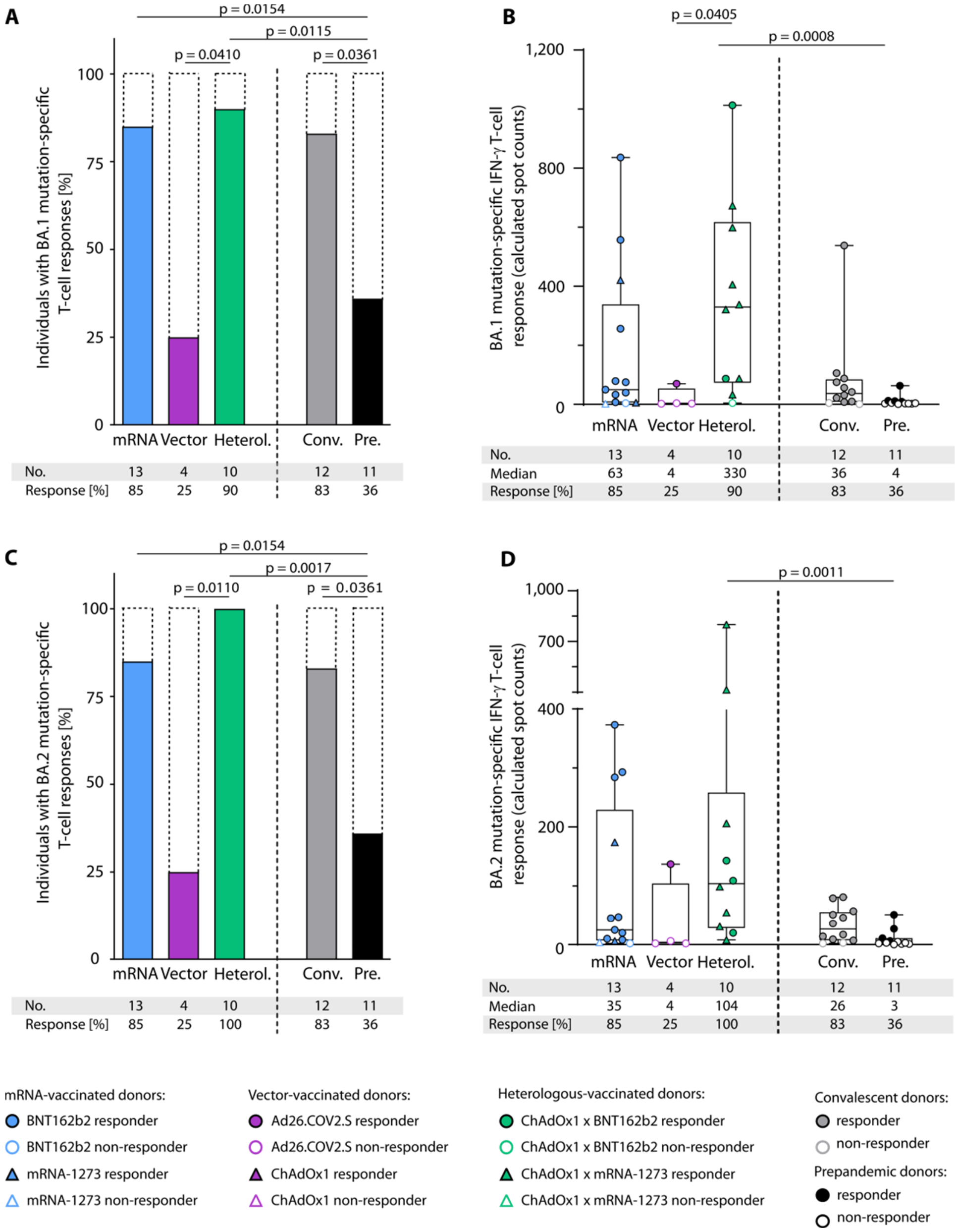

Fig. S4: IFN-γ responses to SARS-CoV-2 BA.1 and BA.2 mutation pools following 12-day *in vitro* expansion. A-D, T-cell responses after complete vaccination (two doses of either BNT162b2, mRNA-1273 or ChAdOx1, one dose of Ad26.COV2.S, or one dose of the vector vaccine ChAdOx1 followed by one dose of an mRNA vaccine for heterologous vaccine regimens), were assessed by interferon-gamma (IFN-γ) enzyme-linked immunospot (ELISpot) assays after 12-day *in vitro* T-cell expansion against the SARS-CoV-2 BA.1 and BA.2 mutation pools. **A,** Percentage of individuals with IFN-γ ELISpot T-cell responses after

12-day expansion, and **B,** intensities of IFN-γ T-cell responses in terms of calculated spot counts against the spike BA.1 mutation pool, after mRNA, vector or heterologous (heterol.) vaccination, compared to COVID-19 convalescents (Conv.) and prepandemic (Pre.) donors. **C,** Percentage of individuals with IFN-γ ELISpot T-cell responses after 12-day T-cell expansion, and **D,** intensities of IFN-γ T-cell responses in terms of calculated spot counts against the spike BA.2 mutation pool. Responders are represented by colored symbols, non-responders by clear symbols. Symbol shapes indicate the different vaccine products received by the donors. **B,D,** box plots represent the median with 25th and 75th percentiles with minimum and maximum whiskers. **A,C,** Fisher's exact test was used. **B,D**, Kruskal-Wallis test was used, if no p values are shown results were not significant. No., number.

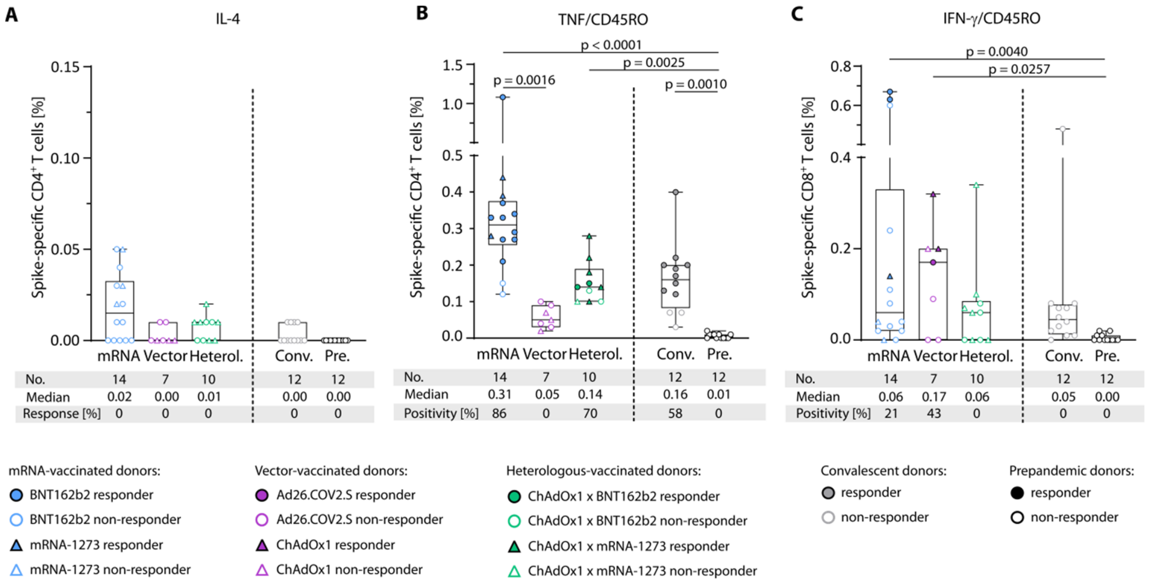

Fig. S5: *Ex vivo* characterization of spike-specific T-cell responses after complete vaccination. A-C, Spike-specific T-cell responses after complete vaccination (two doses of either BNT162b2, mRNA-1273 or ChAdOx1, one dose of Ad26.COV2.S, or one dose of the vector vaccine ChAdOx1 followed by one dose of an mRNA vaccine for heterologous vaccine regimens) were characterized *ex vivo* by intracellular cytokine (interferon-gamma (IFN-γ), interleukin-4 (IL-4), tumor necrosis factor (TNF)) and surface marker (CD45RO) staining. **A**, Frequency of spike-specific IL-4 expressing $CD4^+$ T cells. **B**, Frequency of $TNF^+CD45RO^+CD4^+$ T cells. **C**, Frequency of $IFN\text{-}\gamma^+CD45RO^+CD8^+$ T cells. T-cell responses were considered positive if the detected frequency of cytokine- and surface marker positive $CD4^+$ or $CD8^+$ T cells was ≥ 3-fold higher than the frequency in the negative control and minimum 0.1% of total $CD4^+$ or $CD8^+$ T cells. Responders are represented by colored symbols, non-responders by clear symbols. Symbol shapes indicate the different vaccine products received by the donors. Box plots show the median with 25th and 75th percentiles, whiskers represent minimum and maximum; Kruskal-Wallis test was used, if p values are not shown the results were not significant. FSC, forward scatter; Neg., negative control.

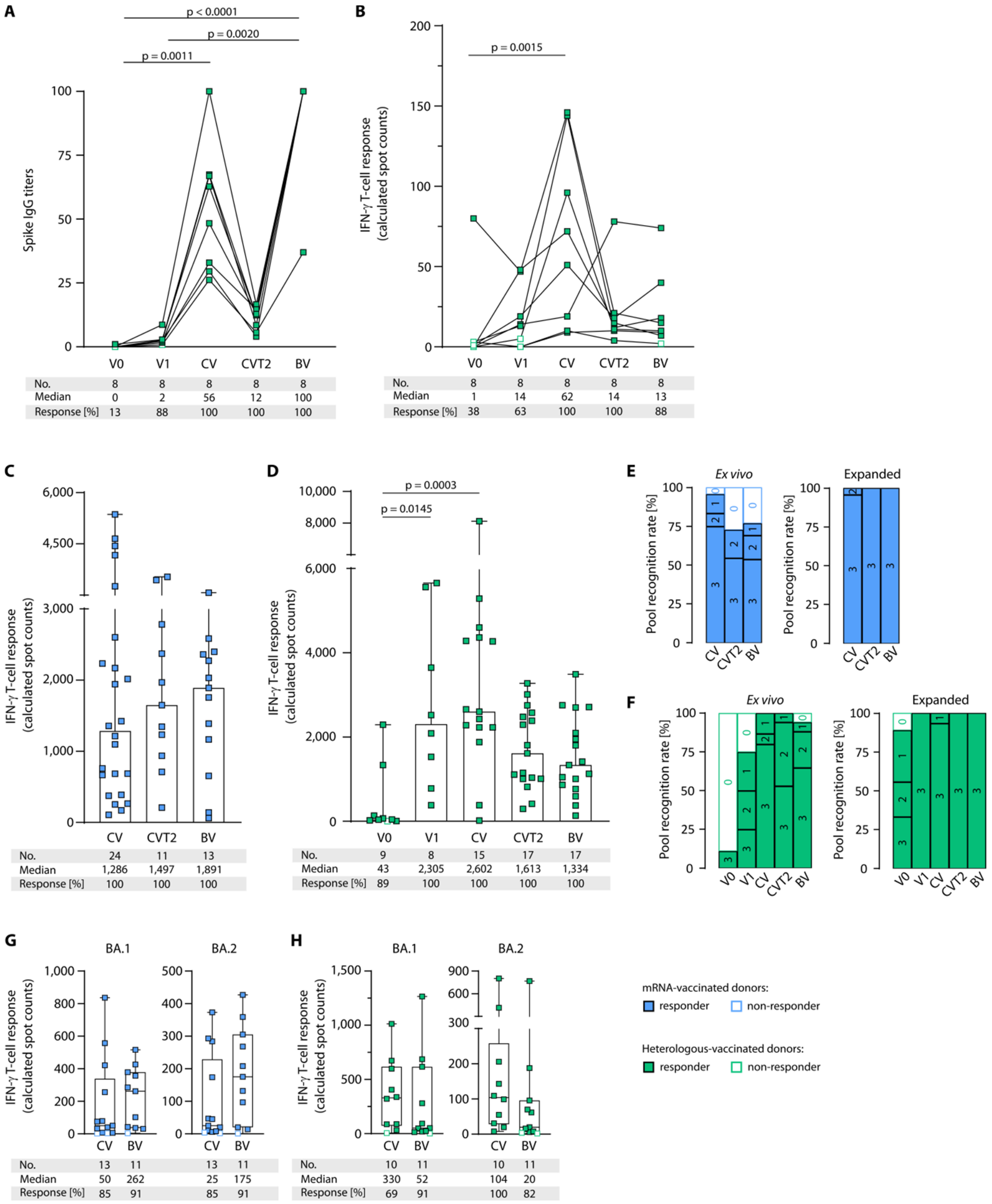

Fig. S6: T-cell responses following mRNA and heterologous vaccination. A, B, Paired spike antibody titers (**A**), and intensities of *ex vivo* interferon-gamma (IFN-γ) T-cell responses in terms of calculated spot counts targeting spike-specific peptide pools (**B**) after heterologous vaccination, either before (V0), one month after first (V1) and complete vaccination (CV), six months after complete vaccination (CVT2) and one month after boost vaccination (BV). **C,D,**

Intensity of interferon-gamma (IFN-γ) T-cell response against the spike peptide pools after 12-day *in vitro* T-cell expansion following mRNA (**C**) and heterologous (heterol.) (**D**) vaccination. **E,F,** Proportion of mRNA- (**E**) or heterologous-vaccinated (**F**) individuals with responses *ex vivo* and after 12-day T-cell expansion to all three, two, one or none of the spike peptide pools (Prot_S1, Prot_S+, Prot_S). **G,H,** Intensities of IFN-γ T-cell responses against the SARS-CoV-2 BA.1 and BA.2 spike mutation pools, after CV and BV for mRNA- and heterologous-vaccinated donors, respectively. Responders are represented by colored symbols, non-responders by clear symbols. **A,B,** Data are presented as scatter dot plots with median, whiskers show maximum. **A,B,** Friedman test was used, **C,D,G,H,** Kruskal-Wallis test was used. No., number.

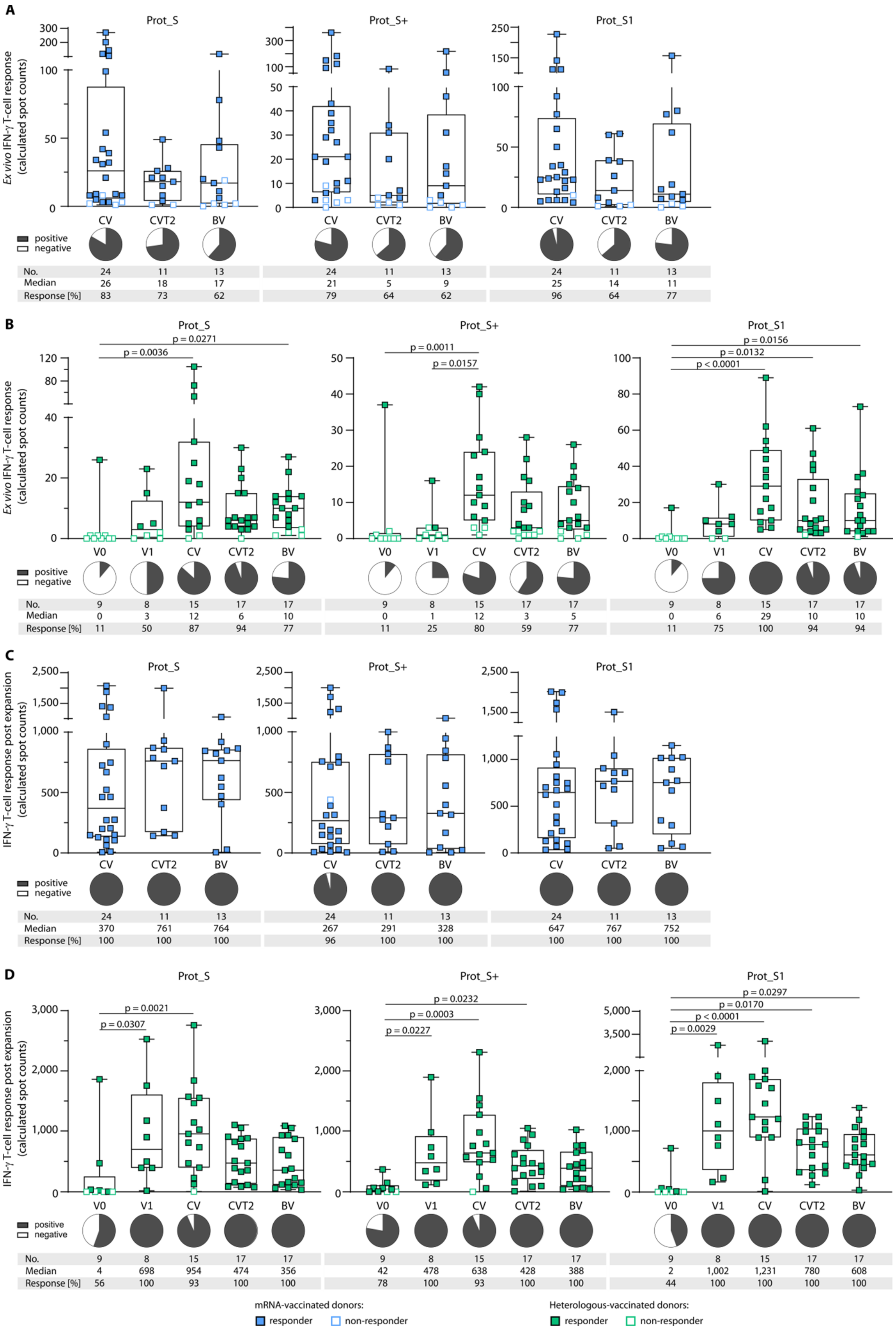

A

	Prot_S CV	Prot_S CVT2	Prot_S BV	Prot_S+ CV	Prot_S+ CVT2	Prot_S+ BV	Prot_S1 CV	Prot_S1 CVT2	Prot_S1 BV
No.	24	11	13	24	11	13	24	11	13
Median	26	18	17	21	5	9	25	14	11
Response [%]	83	73	62	79	64	62	96	64	77

B

	Prot_S V0	Prot_S V1	Prot_S CV	Prot_S CVT2	Prot_S BV	Prot_S+ V0	Prot_S+ V1	Prot_S+ CV	Prot_S+ CVT2	Prot_S+ BV	Prot_S1 V0	Prot_S1 V1	Prot_S1 CV	Prot_S1 CVT2	Prot_S1 BV
No.	9	8	15	17	17	9	8	15	17	17	9	8	15	17	17
Median	0	3	12	6	10	0	1	12	3	5	0	6	29	10	10
Response [%]	11	50	87	94	77	11	25	80	59	77	11	75	100	94	94

C

	Prot_S CV	Prot_S CVT2	Prot_S BV	Prot_S+ CV	Prot_S+ CVT2	Prot_S+ BV	Prot_S1 CV	Prot_S1 CVT2	Prot_S1 BV
No.	24	11	13	24	11	13	24	11	13
Median	370	761	764	267	291	328	647	767	752
Response [%]	100	100	100	96	100	100	100	100	100

D

	Prot_S V0	Prot_S V1	Prot_S CV	Prot_S CVT2	Prot_S BV	Prot_S+ V0	Prot_S+ V1	Prot_S+ CV	Prot_S+ CVT2	Prot_S+ BV	Prot_S1 V0	Prot_S1 V1	Prot_S1 CV	Prot_S1 CVT2	Prot_S1 BV
No.	9	8	15	17	17	9	8	15	17	17	9	8	15	17	17
Median	4	698	954	474	356	42	478	638	428	388	2	1,002	1,231	780	608
Response [%]	56	100	93	100	100	78	100	93	100	100	44	100	100	100	100

Fig. S7: Spike-specific peptide pool recognition during the course of mRNA and heterologous vaccination. A-D, Intensities of interferon-gamma (IFN-γ) T-cell responses in terms of calculated spot counts *ex vivo* (**A,B**) and after 12-day T-cell expansion (**C,D**), reflecting the course of mRNA- (**A,C**) and heterologous (heterol.)-vaccinated (**B,D**) individuals shown for the distinct spike protein peptide pools (Prot_S1, Prot_S+, Prot_S). Responses are shown either before (V0), one month after first (V1) and complete vaccination (CV), six months after complete vaccination (CVT2) and one month after third vaccination (BV). Responders are represented by colored symbols, non-responders by clear symbols. **A-D**, box plots represent the median with 25th and 75th percentiles with minimum and maximum whiskers. **A-D,** Friedman test was used. No., number.

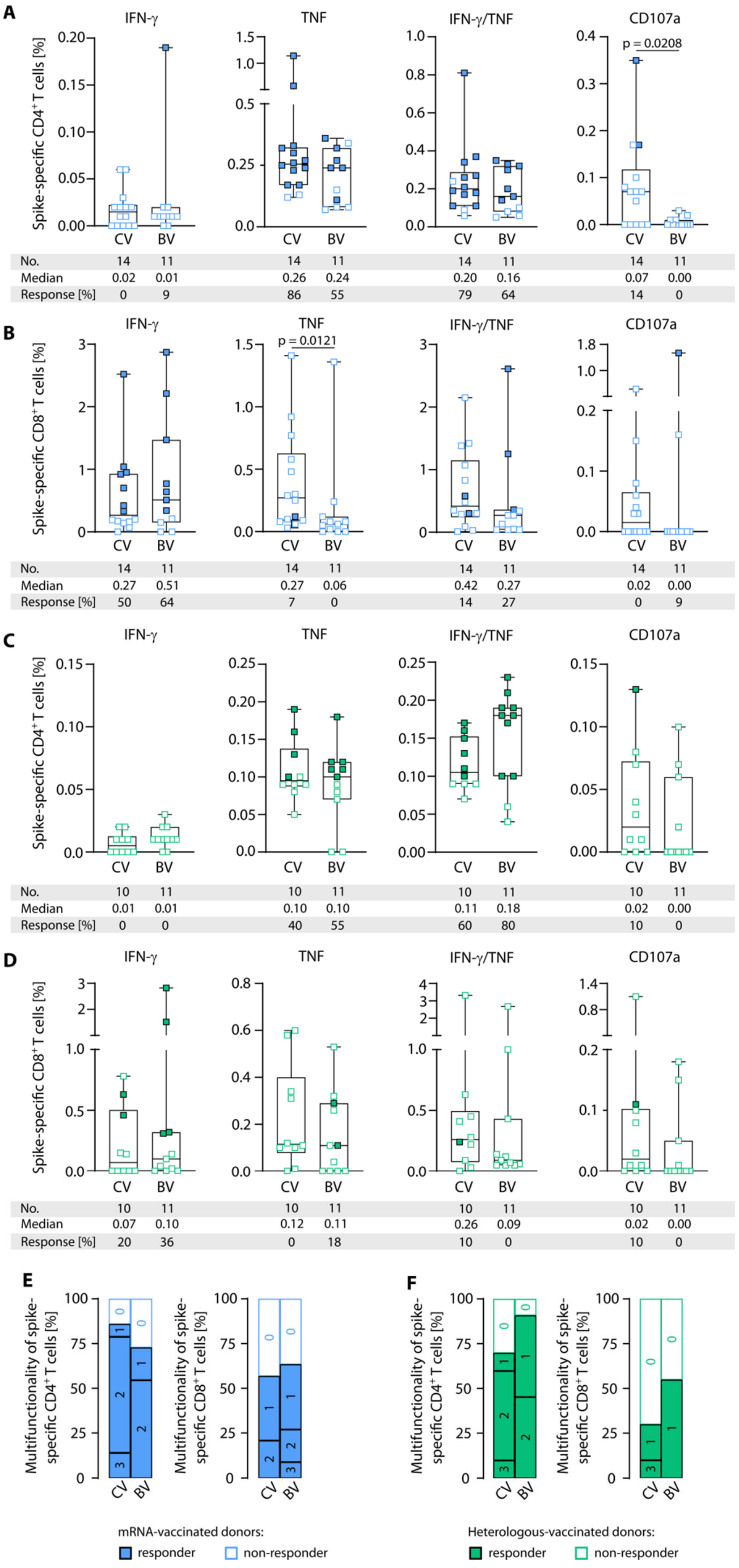
A
IFN-γ
TNF
IFN-γ/TNF
CD107a
Spike-specific CD4+ T cells [%]
p = 0.0208
CV BV
No. 14 11 14 11 14 11 14 11
Median 0.02 0.01 0.26 0.24 0.20 0.16 0.07 0.00
Response [%] 0 9 86 55 79 64 14 0
B
IFN-γ
TNF
IFN-γ/TNF
CD107a
Spike-specific CD8+ T cells [%]
p = 0.0121
CV BV
No. 14 11 14 11 14 11 14 11
Median 0.27 0.51 0.27 0.06 0.42 0.27 0.02 0.00
Response [%] 50 64 7 0 14 27 0 9
C
IFN-γ
TNF
IFN-γ/TNF
CD107a
Spike-specific CD4+ T cells [%]
CV BV
No. 10 11 10 11 10 11 10 11
Median 0.01 0.01 0.10 0.10 0.11 0.18 0.02 0.00
Response [%] 0 0 40 55 60 80 10 0
D
IFN-γ
TNF
IFN-γ/TNF
CD107a
Spike-specific CD8+ T cells [%]
CV BV
No. 10 11 10 11 10 11 10 11
Median 0.07 0.10 0.12 0.11 0.26 0.09 0.02 0.00
Response [%] 20 36 0 18 10 0 10 0
E
Multifunctionality of spike-specific CD4+ T cells [%]
Multifunctionality of spike-specific CD8+ T cells [%]
F
Multifunctionality of spike-specific CD4+ T cells [%]
Multifunctionality of spike-specific CD8+ T cells [%]
mRNA-vaccinated donors:
responder
non-responder
Heterologous-vaccinated donors:
responder
non-responder

Fig. S8: Characterization of *ex vivo* spike-specific T-cell responses following complete and booster vaccination with mRNA and heterologous vaccination regimens. Frequencies of spike-specific $CD4^+$ (**A**) and $CD8^+$ (**B**) T cells following complete vaccination (CV , two doses of either BNT162b2 and mRNA-1273) (one dose of the vector vaccine ChAdOx1 followed by one dose of an mRNA vaccine for heterologous vaccine regimens) and boost (BV) mRNA vaccination. Frequencies of spike-specific $CD4^+$ (**C**) and $CD8^+$ (**D**) T cells following CV (one dose of the vector vaccine ChAdOx1 followed by one dose of an mRNA vaccine) and BV with the heterologous vaccination regimen. Frequencies were assessed *ex vivo* by intracellular cytokine (interferon-gamma (IFN-γ), tumor necrosis factor (TNF)) and surface marker (CD107a) staining. T-cell responses were considered positive if the detected frequency of cytokine-positive $CD4^+$ or $CD8^+$ T cells was ≥ 3-fold higher than the frequency in the negative control and at least 0.1%. Responders are represented by colored symbols, non-responders by clear symbols. **A-D**, box plots show median with 25th and 75th percentiles, whiskers represent minimum and maximum. No., number.

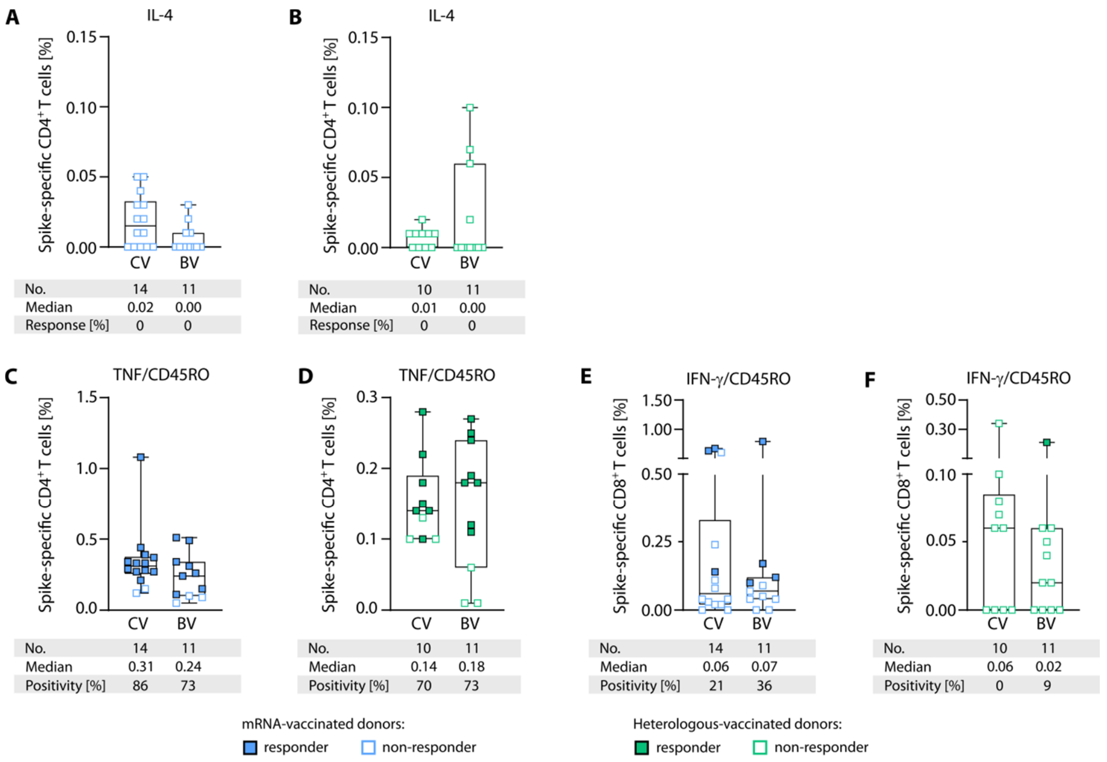

Fig. S9: Characterization of *ex vivo* spike-specific T-cell responses after complete vaccination. A,B, Frequencies of spike-specific IL-4 expressing $CD4^+$ T cells after complete vaccination (CV) and boost vaccination (BV) assessed *ex vivo* using intracellular cytokine and surface marker staining for mRNA- and heterologous-vaccinated donors, respectively. **C,D,** Frequency of $TNF^+CD45RO^+CD4^+$ T cells for mRNA- and heterologous-vaccinated donors, respectively. **E,F,** Frequency of IFN-$\gamma^+CD45RO^+CD8^+$ T cells for mRNA- and heterologous-vaccinated donors, respectively. T-cell responses were considered positive if the detected frequency of cytokine- and surface marker positive $CD4^+$ or $CD8^+$ T cells was ≥ 3-fold higher than the frequency in the negative control and at least 0.1% of total $CD4^+$ or $CD8^+$ T cells. Responders are represented by colored symbols, non-responders by clear symbols. **A-F,** box plots show the median with 25th and 75th percentiles, whiskers represent minimum and maximum. **A-F,** Kruskal-Wallis test was used. FSC, forward scatter; Neg., negative control.

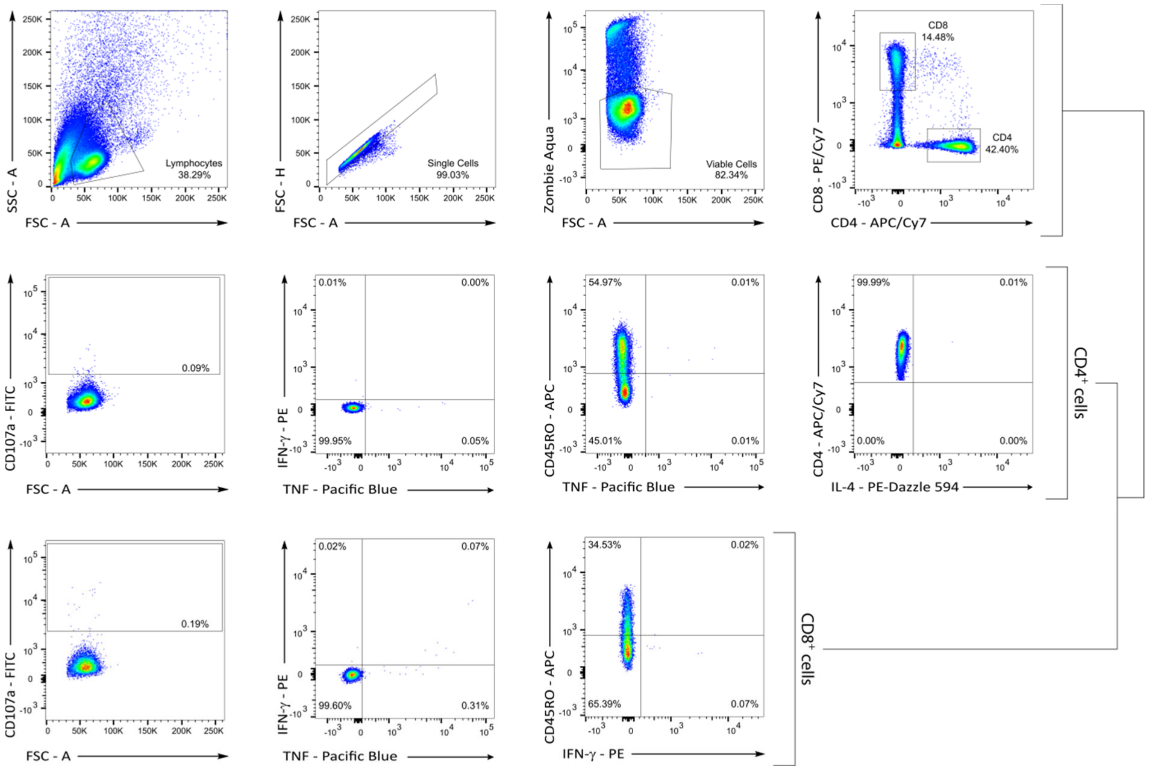

Fig. S10: Gating strategy for *ex vivo* flow cytometry-based evaluation of surface marker and intracellular cytokine staining on a FACS LSRFortessa. Representative example showing the gating strategy for the evaluation of flow cytometry-acquired surface marker and intracellular cytokine staining *ex vivo* data. The first gate identifies the lymphocytes (FSC-A vs. SSC-A), which are further gated for single cells (FSC-A vs. FSC-H) and viable cells (FSC-A vs. Zombie Aqua). Populations of $CD4^+$ and $CD8^+$ T cells (CD4-APC/Cy7 vs. CD8-PE/Cy7) are analyzed separately for the degranulation marker CD107a (FSC-A vs. CD107a-FITC) and different cytokines (TNF-Pacific Blue vs. IFN-γ-PE; CD45RO-APC vs. TNF-Pacific Blue (only $CD4^+$ cells); CD4-APC/Cy7 vs. IL-4-PE-Dazzle 594 (only $CD4^+$ cells), CD45RO-APC vs. IFN-γ-PE (only $CD8^+$ cells)). This gating strategy was applied for the data presented in Fig. 3, Fig. 4G-L, Fig. S8 and Fig. S9A,B.

Table S1: Mutations described for the BA.1 and BA.2 Omicron variants of concern (VOC) affecting the spike protein and spike-derived peptide pools

	Mutated aa total				Mutated aa per 100 aa			
	**Spike protein**	**Prot_S1**	**Prot_S+**	**Prot_S**	**Spike protein**	**Prot_S1**	**Prot_S+**	**Prot_S**
**B.1.1.529.1 (Omicron BA.1)**	34	26	3	12	2.67	3.75	1.45	2.07
**B.1.1.529.2 (Omicron BA.2)**	30	24	2	9	2.35	3.46	0.97	1.55

Number of mutations described for the different variant of concerns within the spike protein (1,274 aa length) and the three spike-derived peptide pools Prot_S1 (693 aa length), Prot_S+ (207 aa length) and Prot_S (580 aa length). aa, amino acids.
